# The cost-effectiveness of physician assistants/associates: A systematic review of international evidence

**DOI:** 10.1371/journal.pone.0259183

**Published:** 2021-11-01

**Authors:** G. T. W. J. van den Brink, R. S. Hooker, A. J. Van Vught, H. Vermeulen, M. G. H. Laurant

**Affiliations:** 1 Radboud Institute for Health Sciences, IQ healthcare, Radboud University Medical Center, Nijmegen, The Netherlands; 2 HAN University of Applied Sciences, School of Health Studies, Nijmegen, The Netherlands; 3 Adjunct Professor, Health Policy, Northern Arizona University, United States of America; Ohio State University Wexner Medical Center Department of Surgery, UNITED STATES

## Abstract

**Background:**

The global utilization of the physician assistant/associate (PA) is growing. Their increasing presence is in response to the rising demands of demographic changes, new developments in healthcare, and physician shortages. While PAs are present on four continents, the evidence of whether their employment contributes to more efficient healthcare has not been assessed in the aggregate. We undertook a systematic review of the literature on PA cost-effectiveness as compared to physicians. Cost-effectiveness was operationalized as quality, accessibility, and the cost of care.

**Methods and findings:**

Literature to June 2021 was searched across five biomedical databases and filtered for eligibility. Publications that met the inclusion criteria were categorized by date, country, design, and results by three researchers independently. All studies were screened with the *Risk of Bias in Non-randomised Studies—of Interventions* (ROBIN-I) tool. The literature search produced 4,855 titles, and after applying criteria, 39 studies met inclusion (34 North America, 4 Europe, 1 Africa). Ten studies had a prospective design, and 29 were retrospective. Four studies were assessed as biased in results reporting. While most studies included a small number of PAs, five studies were national in origin and assessed the employment of a few hundred PAs and their care of thousands of patients. In 34 studies, the PA was employed as a substitute for traditional physician services, and in five studies, the PA was employed in a complementary role. The quality of care delivered by a PA was comparable to a physician’s care in 15 studies, and in 18 studies, the quality of care exceeded that of a physician. In total, 29 studies showed that both labor and resource costs were lower when the PA delivered the care than when the physician delivered the care.

**Conclusions:**

Most of the studies were of good methodological quality, and the results point in the same direction; PAs delivered the same or better care outcomes as physicians with the same or less cost of care. Sometimes this efficiency was due to their reduced labor cost and sometimes because they were more effective as producers of care and activity.

## Introduction

Healthcare systems across the globe face several challenges to meet patient demand and deliver high-quality healthcare. The challenges are primarily population growth, increasing chronically ill patients, rising patient expectations, and longevity [1]. At the same time, the supply of physicians is limited in many countries, leading to medical labor shortages [2].

The gap between physician capacity and patient demand is expanding and requires a change to the medical workforce. At least 18 countries have introduced the Physician Assistant/Associate (PA) as a solution [3]. These health professionals perform various medical and surgical services, and their numbers are growing across multiple settings.

In labor economics, if a PA replicates the activities of a physician, then that is a ‘physician substitute’ [4]. If, on the other hand, the PA improves the throughput in the medical process, then the PA is a ‘complement’ of physician services [5, 6]. In most instances, the employment of the PA was the result of a medical shortage or a need to improve the quality of the medical service.

Because of their increasing utilization worldwide, understanding the economic value has become essential to their utilization. To date, no published systematic reviews have examined the cost-effectiveness of the PA. Therefore, the aim of this project was to review the effects of quality of care, accessibility of care, and costs of physician substitution by PAs in a variety of settings. This was operationalized as a research question: *What is the cost-effectiveness of PAs compared to physician services*?

## Methods

A systematic review was undertaken using the reporting criteria developed at the University of York [7]. The protocol outlined the overview, objectives, aims, operational definitions, search strategy, inclusion/exclusion criteria, and quality appraisal methods.

### Search strategy

The following international bibliographies were systematically searched: PubMed, Web of Science core collection (WoS), CINAHL (with full-text EBSCO), Embase-Ovid, and The Cochrane Library. A detailed search strategy was developed in consultation with two experts; a librarian experienced in systematic reviews and a health workforce researcher. The search strategy used PubMed as a format and then adapted it to the other database results. Searches were performed in 2021 and spanned all published studies through June 2021. Subsequently, the included articles and references were examined using a backward and forward snowball citation search method in Web of Science and Google Scholar to identify relevant other studies.

### Inclusion and exclusion criteria

The literature search included all original empirical research studies on PAs with a comparative quantitative evaluation design written in English or Dutch. There were no date restrictions on publications. Both ‘Physician Assistant’ and ‘Physician Associate’ were included in the review, as they have a similar scope of practice. In addition, studies of ‘Clinical Assistants’ working in South Africa were included because their role is similar to, and modeled after, the PA [8, 9].

Studies that encompassed nurse practitioners (NPs) and PAs but the provider type was missing were excluded. We omitted findings in which PAs were still in training, or the setting had an educational purpose. Articles were excluded when the outcome of care did not fit the protocol or where the care outcome of PAs was not compared to those of physicians.

### Study selection

Citations from the systematic literature search were uploaded to the screening process to *Rayyan QCRI*, a systematic review computer-based application system [10]. Two of three reviewers screened all articles independently (GvdB, AvV, RSH) and were blinded to the others’ findings. Abstracts were vetted using the inclusion/exclusion criteria, and ineligible reports were omitted. Those abstracts receiving conflicting votes were discussed, and after reading the text, consensus for inclusion or exclusion was reached. Articles were rejected when a PA and NP were included in the aggregate but not separated as two providers (and not compared one to the other).

### Data collection, analysis, and synthesis

Two reviewers (GvdB, RSH), acting independently, extracted data from each article using a structured form and blinded to the other’s findings. In addition, five corresponding authors of a candidate study were asked for clarifying information, such as the number of PAs in the project or how many clinics were involved.

Each article was assessed for quality using the *Risk of Bias in Non-Randomised Studies-of Interventions* (ROBIN-I) tool. The ROBIN-I instrument was developed for healthcare evaluation with potential biases in non-randomized studies that compare the effects of two or more interventions [11]. Assessing the risk of bias resulted in a summary score for every research domain ranging from 0 when there was no information; 1 for low risk of bias; 2 for moderate risk of bias; 3 for a significant risk of bias; and 4 for risk of bias was critical. When there was no information, the score was assessed as a serious risk of bias. These different scores per domain result in an overall risk of bias score from 1 to 4 (low bias to the critical risk of bias).

The first 19 data-extracted articles were reviewed by two reviewers independently, and a 97% agreement was reached for all criteria. Based on the high degree of agreement, the remaining articles were assessed by one reviewer (GvdB). The different scores per domain resulted in an overall risk of bias from 1 to 4 (low to critical risk of bias).

Extracted data were organized as:

1. General information (i.e., author, year of publication, country, setting).
2. Study design, follow-up period, research question.
3. Description of the intervention and whether the PA acted as a labor substitute or complement to a physician.

Papers that draw on the same study were extracted and analyzed as one study.

The following outcomes representing cost-effectiveness were assessed:

#### Quality of care

The quality measurement of healthcare is based on the Donabedian model [12]. Metrics of quality of care are *outcomes of care* and the *process of care*. Evaluating the quality of care underpins the measurement for organizational improvement and is a primary focus of health services research [13].

*Patient outcomes*. these include morbidity, mortality, patient satisfaction, quality of life, health status, knowledge, and preference for a physician or PA.

*Process of care outcomes*. patient safety, quality of healthcare, adherence/compliance to guidelines or protocols, healthcare activities (examination, provision of advice, etc.), and referrals to other healthcare services.

*Care provider (physician*, *PA) outcomes*. includes workload (objective and subjective) and job satisfaction.

#### Accessibility of care

The focus on the accessibility of care is the employment effect of the PA on a patient entering the healthcare system. A component of access is the patient’s waiting time to be seen for a medical or surgical condition.

#### Costs of care

*Cost of care* is the expenditures or utilization of resources in the delivery of healthcare services.

## Results

**Fig 1:**
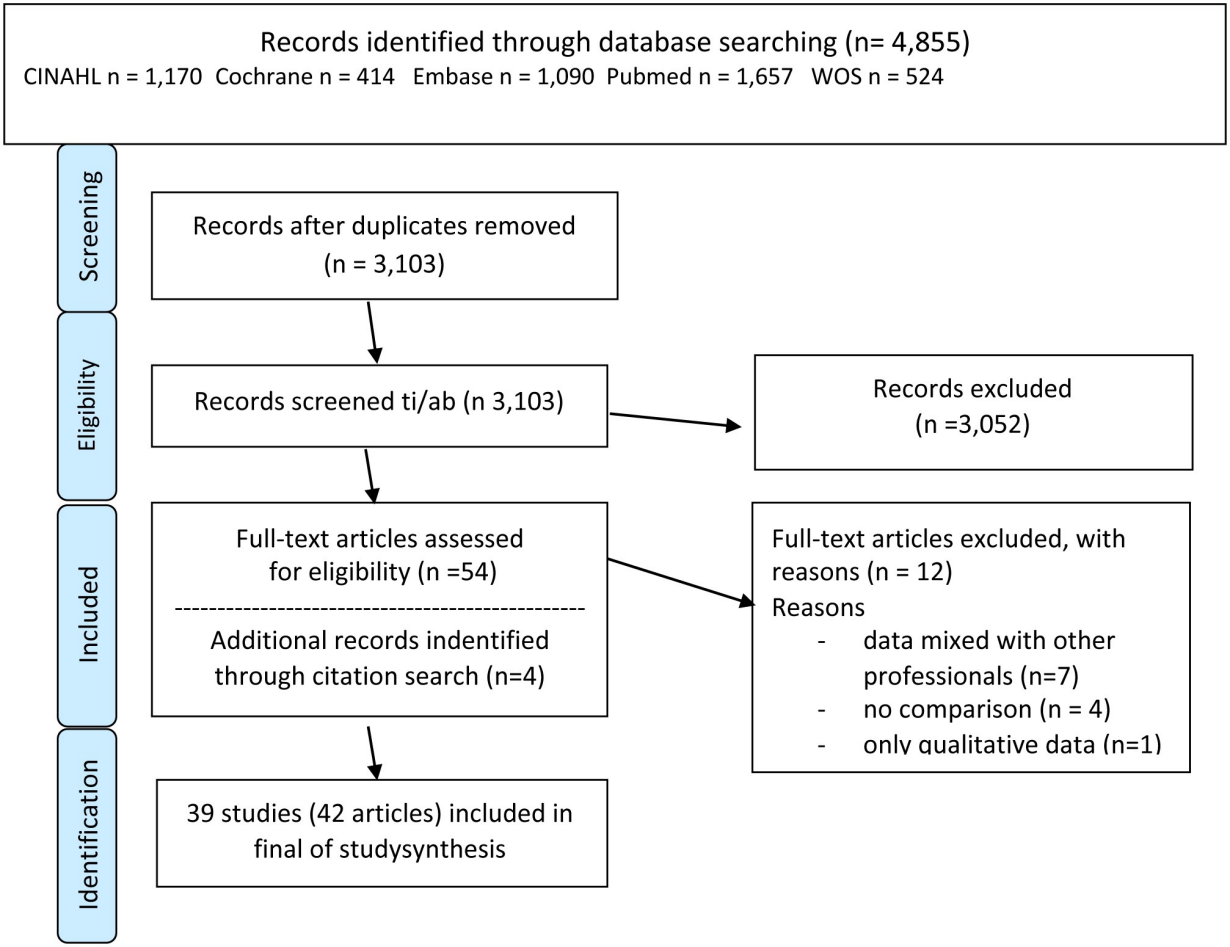
Literature retrieval and study selection.

In total, there were 4,855 titles of abstracts, papers, or reports identified by searching the bibliographies. After de-duplicating, 3,103 titles remained and were screened on title and abstract. The remaining records were assessed for the availability of a full report or article that was peer-reviewed prior to publication. Many titles were poster or presentation abstracts without sufficient details on the methods and analysis and were excluded. After this screening, 54 articles remained, resulting in discussion and five instances of communicating with the author for more information. As a result of the final filtering process and discussion of each paper, a total of 42 articles emerged from the sorting process for final inclusion. The literature retrieval and study selection are shown in Fig 1.

### Characteristics of included studies

The included articles (N = 42) spanned the years 1977–2021. The national origins of the articles were: North America (n = 34), Europe (n = 7), and Africa (n = 1). Over 500 PAs were involved or observed, and their numbers ranged from 1 to 443 (almost 50% of the studies reporting five or fewer). The quantity of PAs in the aggregate is unknown since the number was not consistently stated, and follow-up correspondence with authors did not often reveal more information. Study designs ranged from retrospective cohort studies (n = 29) to prospective studies (n = 10—including one time-motion study).

### Description of the intervention

Throughout the studies, the effect of the introduction of PAs into the medical workforce was described. In most studies (n = 34), the economic labor effect was substitution–*i*.*e*., the PA produced a service that was (or had been) traditionally performed by a physician. The other five studies described a more complementary labor role where the PA enhanced the physician’s role or improved the throughput of medical services [14–18].

Eight studies described the introduction of a PA for a single procedure [17–24]. In the remainder of the studies, the PA was assigned broad medical tasks. In five studies, the introduction of the PA was accompanied by organizational changes or the adaptation of various work processes [16, 17, 25–27]. Changes included extra training or expanded time per patient, dedication to some procedure, more supervision by senior medical staff, or a combination of factors.

Ten studies occurred in an emergency department/acute care setting [14–16, 28–34]. Eight were in the Department of Veterans Affairs, Veterans Health Administration [a national setting of 170 large medical centers and 1,400 outpatient clinics in the USA] [19, 35–41]. The remaining studies were in different settings in hospitals.

The characteristics of included studies are listed in Table 1.

**Table 1:** Characteristics of PA cost-effectiveness studies by the first author, setting, number of PAs & design.

First author and Year of Publication (Country)	Setting	Number of PAs involved	Design
Althausen 2013 (USA) [19]	Hospital-based Emergency Department.	2	Design: Retrospective cohort case series assessed the presence or absence of PA. Charts reviewed were of adult patients presenting to an ED in 2005.
			Question: What is the true impact of hospital-based PAs on orthopedic trauma care at a level II community hospital?
			Intervention: 310 patients with orthopedic injuries who received care from a PA.
			Control: 687 patients with orthopedic injuries who received care from an MD.
Arnopolin 2000 (USA) [29]	Hospital-based Emergency Department	5	Design: Retrospective cohort study. Comparison of PAs and physicians [5 PAs and 25 MDs].
			Question: Are PAs an appropriate option for providing services rendered by physicians in an urban urgent-care facility?
			Intervention: PA was the sole provider for patient encounters; 14 diagnostic groups based on the billed ICD-9 codes (4,256 patients).
			Control: MDs saw the same type of patients (5,345).
Capstack 2016 (USA) [26]	Community Hospital Inpatients—Internal Medicine	6	Design: Retrospective cohort study.
			Question: Can a physician-PA hospital staffed model achieve similar clinical outcomes for inpatients in a community hospital compared to a conventional physician hospitalist staffed model?
			Intervention: A high PA-to physician ratio model (“expanded PA”), with 3 physicians/3 PAs and the PAs rounding on 14 patients a day (35.7% of all visits—6,612 patients).
			Control: Low PA-to-physician ratio model (“conventional”), with nine physicians/two PAs and the PAs assessing nine patients a day (5.9% of all visits—10,352 patients).
Costa 2013 (USA) [17]	Hospital: Transplant Surgery	1	Design: Retrospective cohort study—review of 287 consecutive lung procurements performed by either a PA or MD fellow—spanning 5 years.
			Question: Is a PA a cost-effective, reproducible, and safe alternative for surgical fellows and attending surgeons as the lead donor surgeon for consecutive lung procurements?
			Intervention: A transplant-trained PA is the lead donor surgeon for consecutive lung procurements. (197 cases)
			Control: Transplant (MD)-fellows served as senior donor surgeons. (90 cases)
Decloe 2015 (Canada) [42]	Hospital: Infectious Disease Department	1	Design: Retrospective case-control study.
			Question: Does introducing a PA infectious disease consulting service affect inpatient length of stay (LOS) and mortality rates?
			Intervention: The introduction of a PA in a large urban community hospital in Canada (2010 to 2011) in the infectious disease consult service (3,386 patients).
			Control: The two years of MD use and LOS data before the introduction of the PA (13,493 patients).
De la Roche 2021 (Canada) [30]	Hospital: Emergency Department	1	Design: Retrospective cohort study
			Question: What is the effect of a PA working in a hospital emergency department (ED) on the overall performance of the ED?
			Intervention: With the introduction of the PA in the ED, the PA saw 9,701 patients with the family practitioner (PA group).
			Control: 10,776 patients who visit the ED are seen by a family practitioner (MD-group).
DeMots 1987 (USA) [33]	Hospital: Coronary angiography laboratory	1	Design: Prospective cohort study.
			Question: Is it safe and time-saving when a PA performs cardiac catheterization?
			Intervention: 150 cardiac catheterizations performed by a PA.
			Control: 150 cardiac catheterization performed by 4 cardiology fellows.
Dhuper 2009 (USA) [43]	Hospital, Community General medical floors [ICU, coronary care unit, subacute/ intermediate care unit, and telemetry unit].	23	Design: Prospective, Before—After-study, retrospective case-controlled.
			Question: What effects resulted from replacing medical residents with PAs as hospitalists on patient outcomes in a community hospital?
			Intervention: Care delivered by PAs in a general hospital setting. The PAs functioned as the house staff (5,508 patients).
			Control: Care delivered by residents [MDs] in a general hospital setting (5,458 patients).
Drennan 2014 [25] & de Lusignan 2016 (England) [44]	General practitioner offices	4	Design: An observational study based on prospective data.
			Question: What is the quality of patient consultation outcomes, and what are the costs of same-day consultations (patient encounters) by PAs compared to GPs?
			What is the quality of the patient consultation of physician associates in comparison to that of general practitioners?
			Intervention: PAs in GP offices saw all patient encounters for same-day [urgent] appointments (932 patients).
			Control: GP’s office seeing all patients encounters for same-day (urgent) appointments (1,154 patients).
Everett 2019 (USA) [45]	Veterans Affairs Outpatient clinic	unknown	Design: Retrospective cohort study; data extracted from the Veterans Health Administration electronic health record.
			Question: Are there differences in diabetes outcomes between patients (n = 609,668) with different types of primary and supplemental providers (physicians, PAs and NPs)?
			Intervention: Care delivered by PA (n = 24,250) as primary care provider (PCP) and care delivered by PA with physician (n = 14,342).
			Control: Care delivered by: Physician as PCP (n = 408,009) or Physician as PCP plus NP supplemental (n = 39,861) or Physician as PCP plus PA supplemental (n = 24,692). NP as (n = 66,042) or by NP as PCP with physician supplemental (n = 32472)
Faza 2018 (USA) [35]	Veterans Affairs Medical Centers [multiple sites]	409	Design: Retrospective; regression analyses of patients with diabetes or cardiovascular disease (CVD) with a primary care visit in 130 Veterans Affairs Medical Centers to assess the association between provider type and effectiveness of resource use.
			Question: What is the effectiveness of CVD and diabetes care delivered by PAs and NPs in a primary care setting?
			Intervention: Care delivered by PAs (N = 409)
			Control: Care provided by NPs (N = 1,325).
Fejleh 2020 (USA) [19]	Veterans Affairs Medical Center [St. Louis, MO], gastroenterology clinic	5	Design: Retrospective cohort study randomly assigned colonoscopy to PA or MD in a single-center gastroenterology suite.
			Question: What are the differences in quality measures of PAs and MDs in screening colonoscopies?
			Intervention: Quality of 169 procedures by 5 gastroenterology PAs.
			Control: Quality of 428 procedures by 39 MD (Gastroenterologist (7) and fellows (32))
Fung 2020 (USA] [34]	Rural hospital, intensive care unit.	1	Design: Retrospective cohort study.
			Question: What are the effects of adding a PA to the internist-ICU team on mortality, readmission, ICU and hospital LOS, Hospital Intensity Group weighting, and quality of chart documentation?
			Intervention: Adding a PA to the internist ICU team (132 patients).
			Control: An internist ICU team without a PA (136 patients).
Glotzbecker 2013 (USA) [46]	Inpatient academic medical center oncology unit	2	Design: Retrospective cohort study; data collected on all patients with acute myelogenous leukemia (AML) admitted to the house officer or PA working on the hematologic malignancy service for reinduction of chemotherapy from 2008 through 2012.
			Question: What is the quality of AML care provided by an oncology PA compared with an oncology MD?
			Intervention: 48 patients with AML (50.5%) admitted to the PA service.
			Control: 47 patients with AML (49.5%) were admitted to the physicians in training (MD house officers).
Goldman 2004 (USA) [21]	Outpatient surgical abortion services	6	Design: Prospective cohort study of women undergoing a surgically induced abortion. Ninety-one percent of eligible women (1,363) were enrolled.
			Question: What are the complications after surgical abortion procedures performed at two clinics? Also addressed access to abortion services, patient’s and practitioner’s care experiences, and practitioners’ conformance to clinical guidelines.
			Intervention: One hospital at which PAs performed surgical abortions.
			Control: One hospital at which physicians performed abortions.
Grzybicki 2002 (USA) [47]	Family/general medicine practice	1	Design: Retrospective mixed methods, quantitative study concerning the daily activities and the economic effects of employing a PA instead of an MD (13,000 visits).
			Question: What is the economic benefit of PA in a solo medical practice?
			Intervention: Employment of one PA.
			Control: Published national statistics on MD employment.
Halter 2020 (England) [31]	Emergency Departments (3)	6	Design: Retrospective cohort study; charts reviewed and interviews [mixed methods] assessed the clinical adequacy and quality of care.
			Question: Compared to MDs, what is the rate of unplanned return to the emergency departments (EDs) when managed by PAs?
			Intervention: Six PAs working in 3 EDs (1,129 patients).
			Control: 40 foundation doctors (MBBS year 2) working in 3 EDs (2,068 patients).
Hooker 2002 (USA) [48]	Large multi-specialty ambulatory health maintenance organization (HMO)	43	Design: Retrospective, cost-benefit analysis—comparison of providers (PAs or MDs) managed episodes of care and the use of resources for that care. Random selection of patients to PA or MD for an acute condition. No cross-over or shared care. Use of resources was assigned institutional costs.
			Question: Do PAs negate their cost-effectiveness by using more resources for an episode of disease?
			Intervention: Eight clinics where an episode of acute care was managed by PAs longitudinally.
			Control: Eight clinics where an episode of acute care was managed by MDs longitudinally.
Hooker 2004 (USA) [49]	Medium size occupational & environmental medicine (OEM) clinic (8 sites).	12	Design: Retrospective, cost-benefit analysis—comparison of cost of care between MD and PA spanning one year.
			Question: How do PAs & MDs in OEM compare in the outcomes of care?
			Intervention: Clinics where an episode of acute care was managed by 12 PAs longitudinally.
			Control: Clinics where an episode of acute care was managed by 24 MDs longitudinally.
Jackson 2018 (USA) [36]	Department of Veteran Affairs primary care facilities (multiple sites)	443	Design: Retrospective cohort study. The relationship between the PCP (primary care provider the patient most often visited) and the continuous and dichotomous control of hemoglobin A1c, systolic blood pressure, and low-density lipoprotein cholesterol was examined based on the mean of measurements.
			Question: What are the differences in chronic disease outcomes among patients managed by physicians, NPs, and PAs as PCPs?
			Intervention: Care was provided by 443 PAs [25,352 patients].
			Control: Care for 343,129 patients was delivered by physicians (n = 3,487) and NPs (n = 1,445).
Kawar 2011 (USA) [50]	Hospital Medical Intensive Care Unit	4	Design: Prospectively Medical Intensive Care Unit (MICU) data on 5,346 patients admitted to a MICU; 3,971 patients admitted to an MD-managed MICU (resident group) and 1,375 to a PA-managed MICU (PA group).
			Question: What are clinical outcome differences between patients admitted to a resident and a PA MICU?
			Intervention: A 16-bed MICU run by a team of four PAs, a critical care MD fellow, and an attending critical care physician.
			Control: A 32-bed MICU run by two teams consists of 4 to 6 second-year internal medicine residents, a critical care MD (fellow), and an attending critical care physician.
Krasuki 2003 (USA) [22]	Hospital Cardiac Catheterization Lab	3	Design: Retrospective cohort study.
			Question: Is there a difference in the outcomes of patients undergoing cardiac catheterization procedures by PAs vs. MDs?
			Intervention: In total 929 cardiac catheterizations were performed by three supervised PAs.
			Control: MD = 4,521 catheterizations performed by 21 different cardiology fellows with similar supervision.
Kuo 2013 (USA) [51]	Nursing Homes (multiple sites)	Unknown	Design: Retrospective cohort study of 12,249 nursing home (NH) residents managed by PAs or MDs. Potentially avoidable hospitalizations and Medicare costs were assessed, ranging from 6–48 months. Three primary care providers managed NH care (PCPs): 5% PAs, 25% NPs, and 70% MDs.
			Question: Are potentially avoidable hospitalizations of NH residents a function of the percentage of clinical effort their PCP devotes to NH practice?
			Intervention: PAs who worked as the PCP for residents in an NH.
			Control: Physicians who worked as the PCP for residents in an NH.
Malloy 2021 (USA) [24]	Hospital, surgery	1	Design: Retrospective cohort study
			Question: What are the indirect costs in training surgical residents by comparing the differences in operative time and procedural charges between a resident and a PA first-assisting adolescent reduction mammaplasty?
			Intervention: The PA (1) with two years of experience was the first assist surgeon involved in 25 operations.
			Control: A range of residents (15 MDs) served as the first assist surgeon in 24 operations. The remaining surgeons were part of an integrated plastic surgery training program.
Morgan 2008 (USA) [37]	Outpatient clinics: Department of Veterans Affairs: 150 medical centers (national represented data)	Unknown	Design: Retrospective cohort study; data extracted from the Medical Expenditure Panel Survey.
			Question: Is PAs substantive inclusion in patient care associated with increased numbers of office visits per patient, adjusting for case-mix differences between patients seen by PAs and physicians?
			Intervention: A group of patients had a substantive portion (30%) of their office-based visits attended solely by a PA (1,762 adults).
			Control: A group of patients group who received only physician care (111,184 adults).
Morgan 2019 (USA) [38]	Outpatient clinics: Department of Veterans Affairs: 150 medical centers [national represented data]	2,806	Design: Retrospective cohort study; data extracted from the Veterans Health Administration electronic health record.
			Question: What are the healthcare use and the total costs of care among 47,236 medically complex patients veterans with diabetes, comparing physician, NP, and PA primary care providers?
			Intervention: Care delivered by PAs as care providers (2,806).
			Control: Care delivered by physicians as a care provider (36,894).
Nestler 2012 (USA) [15]	Hospital Emergency Department	1	Design: Prospective, observational cohort controlled before-and-after study design.
			A total of 724 adult patients were included. Data were extracted from the medical records.
			Question: Does the employment of a PA, acting as a triage liaison provider (TLP), shorten the LoS and reduce the proportion of patients who ‘leave without being seen?
			Intervention: Spanning 8 pilot days, a PA TLP was added to the existing staffing (371 patients).
			Control: A total of 8 control days without a TLP (335 patients).
Ngcobo 2018 (South Africa) [23]	Surgical Clinic	Unknown	Design: The retrospective analysis consisted of measuring and comparing the presence of adverse events associated with adult circumcisions.
			Question: Do Clinical Associates (CAs) perform circumcisions at a comparable clinical standard as doctors?
			Intervention: 4195 patients operated on by CAs.
			Control: 543 patients operated on by a physician.
Oswanski 2004 (USA) [32]	Emergency Department (Level 1 Trauma Center)	Unknown	Design: Retrospective analysis of patient care for two 6-month segments was at a Level II Trauma Center.
			Question: To assess the quality of patient care during the transition from resident- to PA-assisted trauma program (without residents) and simultaneous comparative support.
			Intervention: 479 patients received care from PAs in a PA-assisted trauma program (without residents) and simultaneous comparative support.
			Control: 293 patients received care from MD resident-assisted trauma program and simultaneous comparative support.
Pavlik 2017 (USA) [33]	General Community Emergency Department—Pediatric Patients	8	Design: Prospective cohort study. During a 24-month study period, a total of 10,369 pediatric patients (0 and 6 years) were treated in the ED. Three different treatment groups were defined for the analysis: emergency physicians (EPs) alone, PAs alone, and PAs with consults from emergency physicians (PA & EP).
			Question: What are the 72-hour recidivism rates of PA-managed pediatric patients in a general emergency department?
			Intervention: PAs alone (2,789 patients) and PA & EP (984 patients) treat young children in an emergency department.
			Control: EPs who alone treat young children (293 patients) in an emergency department.
Resnick 2016 (USA) [18]	Outpatient Oral and Maxillofacial Surgery	2	Design: Prospective cohort study (before-after) of patients from the Department of Plastic and Oral Surgery at a children’s hospital who underwent removal of 4 impacted third molars with intravenous sedation in an outpatient facility. A total of 50 patients, each cohort contained 25 patients.
			Question: What are the time, cost, and complication rates of integrating PAs into the procedural components of an outpatient oral and maxillofacial surgery practice?
			Intervention: Introduction of a PA in the operating team. The PAs obtained procedural consent, provided local anesthesia after adequate intravenous sedation had been delivered, and performed wound closure after removing the third molars by the maxillofacial surgeons.
			Control: A traditional team without PAs.
Roy 2008 (USA) [52]	Academic Medical Center General medicine	5	Design: Retrospective cohort study of 5,194 patients on a general medicine service of a 747-bed Academic Medical Center.
			Question: How is the quality and efficiency of patient care of a PA hospitalist service compared with that of traditional MD house staff services?
			Intervention: Patients (992) were admitted to the general medical service on the PA hospitalist service.
			Control: Patients (4,202) were admitted to the general service with a traditional house staff service.
Singh 2011 (USA) [53]	Academic Medical Center; General Medical Inpatient Care	2	Design: Retrospective study of 9,681 general medical hospitalizations.
			Question: What are the outcomes of inpatient care provided by a hospitalist-PA model compared with the traditional resident-based model?
			Intervention: Hospitalist-PA model for general medical hospitalizations (2,171 patients).
			Control: Traditional resident-based MD model for general medical hospitalizations (7,510 patients).
Smith 2020 (USA) [39]	Outpatient clinics: Department of Veterans Affairs: 170 medical centers (national represented data)	443	Design: Retrospective study of 368,481 adult diabetes patients.
			Question: What are the utilization costs of care by MD, PAs, and NPs?
			Intervention: PA delivered care (25,352 patients).
			Control: MD and NP delivered care (301,361 patients).
Theunissen 2014 (NL) [16]	Academic Medical Center; Emergency Department	2	Design: Prospective comparative intervention design.
			Question: Does the use of a PA in an emergency department’s fast-track (FT) unit have a favorable effect on waiting times and turnaround times?
			Intervention: The group of 1,280 patients was seen at the FT unit by the PA.
			Control: 1,378 patients were seen at the trauma unit by the trainee surgeon.
Timmermans 2017 (a, b) [41, 56] & Bos 2018 (NL) [55]	Large urban Hospitals (multicenter)	25	Design: A retrospective, multicenter, matched-controlled study. Patients were assessed for Quality-Adjusted Life Years (QALY).
			Question: What is the cost-effectiveness (cost-utility) of substitution of care from MDs to PAs
			Intervention: MDs and PAs were assigned inpatient care on 17 wards (1,015 patients).
			Control: The traditional model in which only MDs were assigned inpatient care on 17 wards (1,378 patients).
Tompkins 1977 (USA) [14]	Outpatient clinic for acute respiratory or ear problems	5	Design: Prospective, Time-Motion Study.
			Question: How effective is the medical care for acute respiratory ill patients provided by physicians and algorithm-assisted PAs and military medical assistants?
			Intervention: PAs provided care by an algorithm and supervision by an MD (2,149 patients).
			Control: One group of patients received care provided by MDs [389 patients].
			One group of patients received care from algorithm-assisted military medical assistants (3,212 patients).
van Rhee 2002 (USA) [54]	A large community teaching hospital Internal medicine	16	Design: Retrospective cohort study. A total of 5,194 consecutive patients were admitted to the general medical service, including 992 patients on the PA/hospitalist service and 4,202 patients on a traditional house staff service.
			Question: What is the quality and efficiency of patient care on a PA/hospitalist service compared with traditional house staff services?
			Intervention: A medical service staffed with PAs and supervised by MD hospitalists for inpatient general medicine service of a 747-bed academic medical center.
			Control: Traditional house staff (MD)-service.
Yang 2018 (USA) [40]	Outpatient clinics: Department of Veterans Affairs: 150 medical centers (national represented data) care for diabetic patients	240	Design: Retrospective cohort study
			Question: What is the quality of the primary care for patients with diabetes mellitus managed by primary care NPs, PAs, or physicians? (19,238 patients)?
			Intervention: Care delivered by PAs (1,367 patients).
			Control: Care delivered by physicians and NPs. (15,050 & 2,821 patients).

AML: Acute myelogenous leukemia; ED: emergency department; EP: emergency physician; FT: fast track; LoS: Length of Service; MICU: Medical Intensive Care Unit; NL: Netherlands; NPs: nurse practitioners; MDs: medical doctors; PCPs: primary care providers; PAs: physician assistant/associates; QALY: Quality Adjusted Life Years; USA: United States of America; WT: wait times.

*First Author* by last name and year of publication; if a study comprises more than one publication of all papers, first author and year publication is reported. *Setting* is where the study took place. *Number of PAs* was extracted from the publication or communication with an author. *Design* was whether it was randomly controlled, prospective, or retrospective. *Question* was the research question or hypothesis. *Intervention* describes the role of the PA. *Control* describes the part of physician services without a PA.

### Risk of bias in individual studies

**Fig 2:**
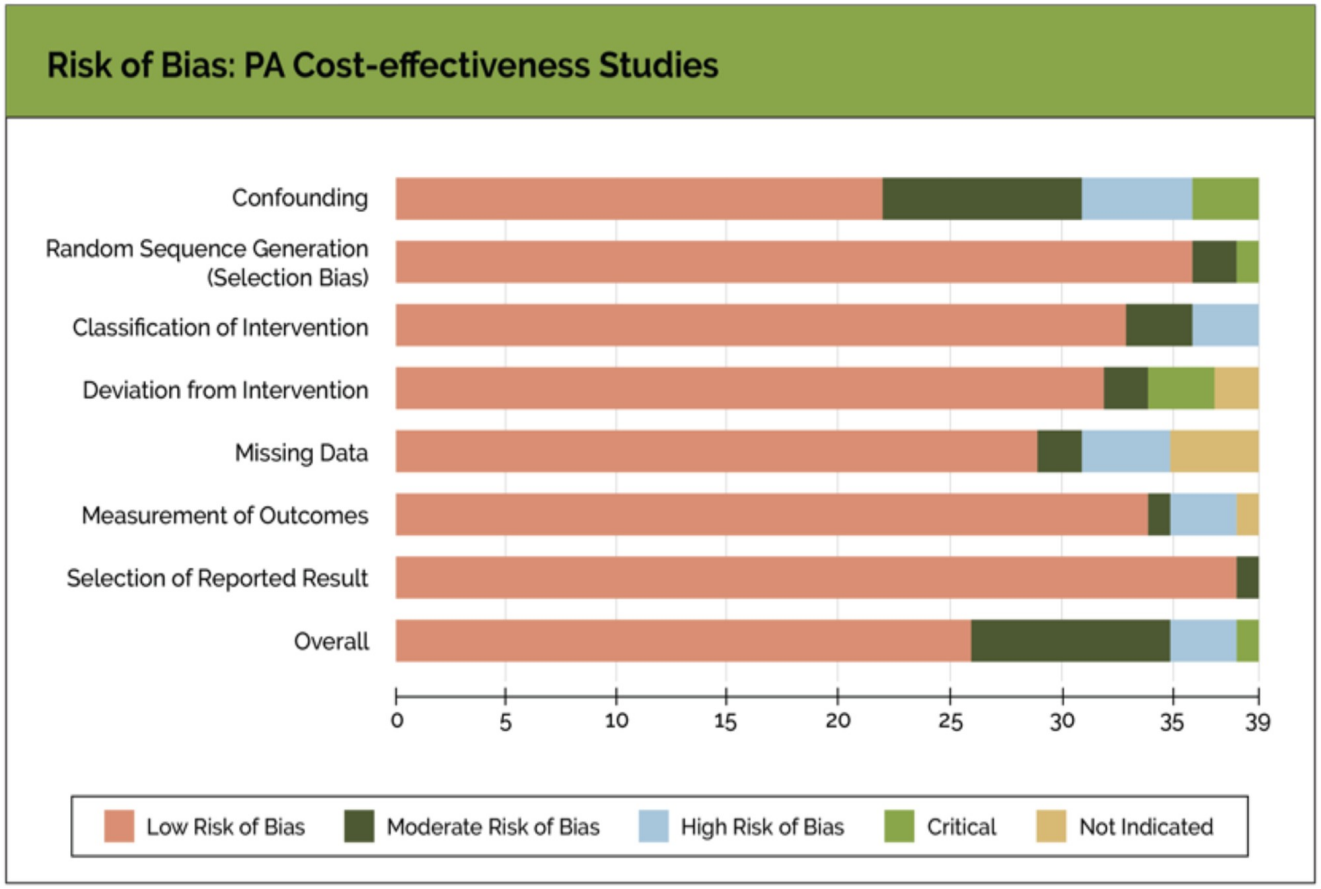
Risk of bias: Cost-effectiveness studies. The risk of bias graph is a summary of the review authors’ judgment about each assessed risk of bias article presented across all studies.

Thirty-five of 39 studies in this review had a low risk of bias when assessed by the selection process, including missing data and results (See S1 and S2 Appendices for details). However, three studies [20, 29, 43] scored a serious risk of bias, and one study [17] scored a critical risk of bias in terms of confounding variables. The risk of bias scores is summarized and displayed in Fig 2.

### Outcomes of care

Outcomes of care studies were assessed for:

- Patient outcomes
- Process of care
- Accessibility of care
- Costs of care

The results are discussed below and displayed in Table 2.

**Table 2:** Outcomes of care are based on the quality of care, accessibility of care, and cost of care.

First author & Year of Publication [reference #]	Quality of Care		Accessibility of care	Costs of care
	Patient outcomes	Process of care outcomes		
Althausen 2013 [28]	Intervention vs. control group No differences in types of surgical complications; use of a PA decreased postoperative complication rates by 4.67% (p = 0.0034)	Intervention vs control group: Use of deep vein thrombosis prophylaxis increased 6.73% (p = 0.0084) Postoperative antibiotic administration increased by 2.88% (p = 0.0302)	Intervention vs. control group: Emergency department patients with orthopedic injuries were seen 205 minutes faster (P = 0.006). Time to surgery improved 360 minutes (P = > 0.03).	Intervention vs. control group: Setup time was only marginally improved by 43 minutes, whereas operative time, time out of OR, and operative complication rates remained unchanged The PA produced time savings for orthopedic surgeons. LoS (days) 7.96 (9.16) vs 8.57 (13.62) P = 0.26620. Emergency department LoS: decreased per patient by 175 minutes (P = 0.0001).
Arnopolin 2000 [29]	NA	NA	NA	Intervention vs. control group: LoV with PA was 8 minutes longer (p = <0.001). LoV was 82 min and total charge $159, which was $8 less than MD charge (p = 0.013)
Capstack 2016 [26]	No statistically significant differences were found in-hospital mortality and readmissions.	NA	NA	Intervention vs. control group: Patient charges was less ($2644 vs $2724); 95% CI 2.66%–4.39%, P < 0.001. LoS and consultant use were not significantly different with PA.
Costa 2013 [17]	Intervention vs. control group: PA procured lung injury rate was 1 of 197 (0.5%) vs 22 of 90 (24%), respectively. Rates for pulmonary graft dysfunction grade 2 and 3 (combined rates of 32.2% (29 of 90) vs 9.6% (19 of 197) in the control group ((p < 0.01)	NA	NA	NA
Decloe 2015 [42]	Intervention vs control group: The proportion of deaths: 0.22 vs 0.26. In the pre- to post-intervention period; the proportion of deaths was 0.051 vs. 0.055. Not statistically significant (P = 0.14)	NA	NA	Intervention vs. control group: average time to consult was 14.3 vs. 21.4 h (P<0.0001). Improved LoS 16.2 days v.s 20.5 days.
De la Roche 2021 [30]	NA	NA	In the PA group, there was a lower average daily ‘left without being seen’ rate (3.4% vs. 5.2%; *P* < .001).	The average LoV was 348.91 minutes for the control group and 313.85 minutes for the intervention group (P < .001).
DeMots 1987 [20]	Intervention. vs. control group: The outcomes (complication rates and mortality) were the same.	NA	NA	Intervention. vs control group: The cardiac catheterization procedure time for the PA and fellows was 41minutes ± 13 minutes and 44minutes ± 18 minutes.
Dhuper 2009 [43]	Intervention vs. control group: All-cause and case mix index—adjusted mortality was 1.94% vs. 2.85% (P ≤ .001). The adverse event cases were 5 vs 9 (P = .29). Readmission rate within 30 days was 64 vs 69 (P = .34). Patient satisfaction was 95% vs 96% (P = 0.33).	NA	NA	NA
Drennan 2014 [25] & de Lusignan 2016 [44]	Intervention vs control group: Patient satisfaction was the same between the intervention and control groups. Most of the patients in the intervention group responded that they would be willing to consult a PA again (87.3%, 192/220), while 4.1% (9/220) preferred to consult a GP.	Intervention vs. control group: No differences in the rates of prescriptions issued (1.16, 95% CI = 0.87 to 1.53, P = 0.31). Patient records of initial consultations of patients (n = 99) in the intervention and control group (n = 145) were judged as appropriate by GPs independent of the study (P<0.001). All consultations were assessed as safe, but GPs (control group) were rated higher in quality. More patients with chronic problems were seen in the control group and significantly more patients presenting for ‘minor problems or symptoms’ were seen in the intervention (PA) group.	NA	There were no significant differences in: Rates of re-consultation (rate ratio 1.24, 95% confidence interval (CI) = 0.86 to 1.79, P = 0.25). Rates of diagnostic tests ordered (1.08, 95% CI = 0.89 to 1.30, P = 0.44), referrals (0.95, 95% CI = 0.63 to 1.43, P = 0.80). The adjusted average consultation time in the PA group was 5.8 minutes longer than in the physician (control) group [95% CI = 2.46 to 7.1; P<0.001]. The cost per consultation in the PA group was lower (£6.22) (95% CI = –7.61 to –2.46, P<0.001).
Everett 2016 [45]	No clinically meaningful differences were observed between the intervention and control group in intermediate diabetes outcomes—also no differences with the PA as a solitary primary care provider.	NA	NA	NA
Faza 2018 [35]	NA	A chronic disease cohort of 185,694 patients was assigned to the control group and 66,217 assigned to the intervention group. Measurements included blood pressure, beta-blockers, statins, antiplatelets, primary or specialty care visits, lipid panels, and the number of stress tests ordered was comparable between groups.	NA	Intervention vs. control group: No differences in using resources between the two groups.
Fejleh 2020 [19]	NA	PAs performed flexible sigmoidoscopies comparably to gastroenterologists. The technical performance and quality metrics of the PA demonstrated higher cecal intubation rates than gastroenterologists. Comparisons of attending physicians and PAs grouped by years of experience did not show differences in performance.	NA	PAs performed superior to GI fellows with regard to intubation time (7.8 min versus 13.2 min, P <0.001) and were found to have a shorter withdrawal time (9.6 min versus 11.5 min). No significant difference was found between the intubation time of PAs and attending gastroenterologists (7.8 min versus 8.8 min, respectively, P = 0.25).
Fung 2020 [34]	Intervention vs. Control Group: The 30-day mortality was lower in the intervention group. (Intervention group: 26.85; control group 42.03, p < 0.07)	Intervention vs control group There was a difference in the quality of the admission note; the intervention group scored better: (score <0.5 28.65%; vs control group < 0.5 56.15%, p<0.003). This quality reflected both admission notes being present, score = 1, and a further 1.0 if it included a family history (0.5) and listed meds/allergies (0.5). Also, the quality medication transfer list score was better by the intervention group (scored on quality on the medication transfer list: intervention 80.19%; control 99.2%, p<0.08).	NA	Intervention vs control group hospital LoS (intervention group median 7 days; control group 5 days, p<0.002) ICU length of stay (intervention group 69 hours; control group 48 hours, p<0.002). No significant differences in hospital readmission (intervention 35.06; control 42.29, p = 0.46)
Glotz-becker 2013 [46]	Mortality between the two groups was not significantly different. The mean number of consults was less in the intervention group: 1.47 vs. 2.11 (P 0.03) for the control group.	Intensive care unit transfers between the two groups were not significantly different.	NA	Intervention group: LoS 30.9 days (P 0.03); 14-day readmission rate zero (P 0.03). Control group: LoS 36.8 days (P = 0.03). The 14-day readmission rate was 10.6% (P = 0.03).
Goldman 2004 [21]	Intervention vs. control group: Total complication rates were 22.0 per 1000 procedures (95% confidence interval (CI) = 11.9, 39.2) vs 23.3 per 1000 procedures (95% CI = 14.5, 36.8) (P = 0.88).	NA	NA	NA
Grzybicki 2002 [47]	NA	NA	NA	PA had a same-task substitution ratio (0.86) as MD and compensation to production ratio of 0.36. Compared with an MD, the annual revenue generated $99,360 (0.56 FTE). Weekly visit rates were the same.
Halter 2020 [31]	Emergency medicine re-admittance rates within 7 days (n = 194 & 6.1%) showed no difference between PAs & MDs (OR 0.87, 95% CI 0.61 to 1.24, p = 0.437).	Almost all patient records were clinically adequate. PAs were evaluated as assessing patients in a similar way to second-year doctors-in-training. If seen by a PA, patients were more likely to receive an X-ray investigation (OR 2.10, 95% CI 1.72 to 4.24, p<0.001) after adjustment for patient characteristics, triage severity of the condition, and statistically significant clinician intraclass correlation.	NA	NA
Hooker 2002 [48]	NA	NA	NA	In total, 262,490 medical office visits were analyzed for acute conditions as longitudinal episodes of care seen solely by a PA or MD. Patient age, health status, and gender were matched. The use of resources was the same for the PA, the MD, and the outcomes were the same. The labor cost of a PA was 40% that of the MD. PAs cost-effectiveness assessed the resources used for a care episode compared to the MD was slightly less.
Hooker 2004 [49]	Duration of patient’s disability (return to work) was shorter by 1.8 days for PA than MD.	OEM PAs assessed patients the same way as OEM MDs.	NA	In total, 80,764 encounters were analyzed for an acute episode of care seen solely by a PA or MD. The injury severity scale, patient age, and gender were matched for both providers. The use of resources was the same, but the number of days for disability was shorter for the PA. PA cost of care is 50% less due to wages.
Jackson 2018 [36]	No clinically significant variation was found among the intervention and control group concerning diabetes outcomes, suggesting that similar chronic illness outcomes may be achieved by physicians, PAs, and NPs equally. The difference in A1c values compared with physicians was -0.05% (95% CI, -0.07% to 0.02%) for NPs and 0.01% (CI, -0.02% to 0.04%) for PAs. For systolic BP, the difference was -0.08 mm Hg (CI, -0.34 to 0.18 mm Hg) for NPs and 0.02 mm Hg (CI, -0.42 to 0.38 mm Hg) for PAs. For LDL-C, the difference was 0.01 mmol/L (CI, 0.00 to 0.03 mmol/L) (0.57 mg/dL (CI, 0.03 to 1.11 mg/dL)) for NPs and 0.03 mmol/L [CI, 0.01 to 0.05 mmol/L) (1.08 mg/dL (CI, 0.25 to 1.91 mg/dL)) for PAs.	NA	NA	NA
Kawar 2011 [50]	Intervention group vs. control group: Renal insufficiency 22% vs 25% (P– 0.05). Cerebrovascular accidents 5.6% vs 4% (P = .02). No in-hospital difference of mortality or intensive care unit mortality between the two groups. Survival analyses showed no difference in 28-day survival between the two groups.	NA	NA	A PA-run MICU produced no significant differences in survivorship compared to a resident-run MICU: Hospital average LoS was similar between the intervention and control group. Medical Intensive Care Unit LoS: There was no difference between the intervention and control group after correcting for confounders.
Krasuski 2003 [22]	Intervention group vs. control group: Complication ratio 0.54%; vs 0.58%.	NA	NA	Intervention group vs control group: procedural times 70.2 minutes (± 32.6 minutes), vs 72.6 (± 35.2 min); P = 0.045 use of fluoroscopic imaging 10.2 minutes (± 6.5 minutes) vs 12,2 minutes (± 9.9 min); P 0.001. No difference in the volume of contrast media was seen between the two groups.
Kuo 2013 [51]	Nursing home residents (patients) with Principal Care Providers (PCPs: MDs, PAs, or NPs) who devoted less than 5% of their clinical effort to nursing home care were at 52% higher risk of potentially avoidable hospitalization than those whose PCPs committed 85% or more of their clinical effort to NHs. Hazard ratio = 1.52, 95% confidence interval = 1.25–1.83.	NA	NA	The annual Medicare spending (cost) was $2,179 higher than the intervention (PA) group in the control group.
Malloy 2021 [24]	NA	NA	NA	Intervention vs. control group: procedures in the control group took 34 minutes longer and were $3,750 more expensive (P < 0.01, both).
Morgan 2008 [37]	Patients in the intervention (PA) group had 16% fewer office-based visits per year than those receiving care in the control (MD) group (P = <0,01).	Patients in the PA group had about 25 percent fewer emergency department visits (p<0.05). The results for hospital outpatient and inpatient settings were not statistically significant.	NA	NA
Morgan 2019 [38]	Patients of PAs were less likely than MDs to incur hospitalization related to their ambulatory care (PA vs. MD OR: 0.92, 95% CI: 0.8446, 0.997).	NA	NA	PAs incurred fewer resources than MDs for the same matched group of chronically ill patients even in expanded roles. Estimated annual medical expenditures of PAs vs MDs: total (inpatient, outpatient, pharmacy) $32,350 vs $34,650. The estimated mean ratio for differences in expenditures is 0.93 (*p*<0.01).
Nestler 2012 [15]	NA	NA	Emergency waiting room times (LoV) were similar between the intervention group and the control group. Intervention group vs. control group: Proportion of patients leaving without being seen was 1.4% vs 9.7% (p < 0.001).	Intervention group vs. control group: Length of visit: 229 vs 270 minutes (95% CI 168 to 303) (p < 0.001) Treatment room times = 151 (92 to 223) minutes vs 187 minutes (p < 0.001).
Ngcobo 2018 [23]	Intervention group vs. control group: Adverse events occurred during circumcisions 7.1% (n = 4195) vs 8,1% (n = 543) (p = 0.385). Recorded pain, bleeding, swelling, infection, and no wound destruction differed between the intervention and control groups.	NA	NA	Intervention group v.s control group: Procedure time 14.63 minutes v.s 15.25 (P = <0.001).
Oswanski 2004 [32]	No differences between intervention and control mortality rates.	Focused analysis showed 100 percent participation in the intervention group (PAs) during the trauma alert compared to 51 percent by MD residents.	NA	Intervention group vs. control group: LoS was 2.54 +/- 4.65 vs 3.4 +/- 5.81 (P = <0,05)LoS (from entry to the ward floor) was statistically reduced by 1 day in the intervention group.
Pavlik 2017 [33]	Intervention group (PA) vs. control group—emergency physician (EP): Return rate 6.8% vs 8.0%. For the PA & EP group, the return- rate was 9.3%. Recidivism (return) rates for the 3 clinical groups were: PA (6.8%), EP (8.0%), and PA & EP (9.3%) (P < 0.03). Patients admitted to the hospital on their return visits for the 3 clinical groups were as follows: PA (0.4%), EP (0.6%), and jointly PA-EP (0.7%) (P = 0.2).	NA	NA	NA
Resnick 2016 [18]	No significant differences were found in postoperative complications.	NA	NA	Intervention group vs. control group: Average total procedure cost decreased by $75.08 (P < .001). The time that the oral and maxillofacial surgeon was directly involved in the procedure decreased on average 19.2 minutes (P < .001).
Roy 2008 [52]	There is no difference in inpatient mortality, readmissions, or patient satisfaction.	There is no difference in the ICU transfers.	NA	There is no difference in the LoS. The total cost of care was marginally lower on the intervention group (adjusted costs 3.9% lower; 95% confidence interval (CI) 27.5% to 20.3%)
Singh 2011 [53]	The risk of readmission at 7, 14, and 30 days and the risk of inpatient death were similar between the intervention and control groups.	NA	NA	Intervention group vs. control group: Hospitalizations were associatedwith a 6.73% longer LoS (P = 0.005) in the intervention group; 3.17 days vs 2.99 days. Costs (charges) difference of 6.45% p = 0.07 $9,390 vs $9,044.
Smith 2020 [39]	NA	NA	NA	Patients of PAs have lower odds of inpatient admission (odds ratio for PA vs. MD 0.92, 95% CI = 0.87–0.97), and lower emergency department use (0.67 visits on average for PAs, 95% CI = 0.56–0.63). This translates into PAs having ~$500–$700 less health care costs per patient per year (P<0.0001) than MDs
Theunissen 2014 [16]	No differences in mortality and complaints between the intervention and control group.	NA	Intervention vs. control: Overall waiting time (median: -41 min) p<0.0001. The median overall LoS was also significantly shorter (-12 min) p<0,0001	NA
Timmermans 2017 [27] & Bos 2018; [55] & Timmer-mans 2017 [56]	Intervention vs control group: QALY gain: +0.02 (95% CI −0.01 to 0.05). Improved patient experiences (ß 0.49, 95% CI 0.22–0.76, p = .001)	There are no significant differences between the intervention and control groups concerning the adherence to guidelines on medication prescribing or other indicators for quality and safety of care.		Intervention group vs. control group: Personnel costs per patient for the provider primarily responsible for medical care on the ward were lower on the wards (−€11, 95% CI −€16 to −€6, p<0.01). A cost difference of €309 per patient (95% CI €29 to €588, p = 0.030) was found in favor of the control group regarding the LoS. Total costs per patient did not significantly differ between the groups (+€568, 95% CI −€254 to €1391, p = 0.175).
Tompkins 1977 [14]	NA	NA	NA	Intervention vs. control: Diagnostic test costs by the PA were less than the MD control group ($4.26 vs. $5.48). (p <0.05). Direct medical care costs were significantly lower: intervention = $12.78 vs control = $16.86.
van Rhee 2002 [54]	No difference with inpatient mortality, readmissions, or patient satisfaction.	No difference in ICU transfers.	NA	The total cost of care was marginally lower on the intervention group (adjusted costs 3.9% lower; 95% confidence interval (CI) −7.5% to −0.3%), but LoS was not significantly different (adjusted LOS 5.0% higher; 95% CI, −0.4% to +10%) as compared with the control group.
Yang 2018 [40]	Median hemoglobin A1c was comparable at diagnosis (6.6%, 6.7%, 6.7%, P > .05) and after 4 years (all 6.5%, P >.05). A1c levels at initiation of the first (7.5%-7.6%) and second (8.0%-8.2%) oral medications for patients of PA and NPs compared with that of physicians was also similar after adjusting for patient characteristics (all P > .05).	NA	NA	NA

AML: Acute myelogenous leukemia; CI: Confidence Interval; ED: emergency department; EP: emergency physician; FT: fast track; LoV: Length of visit; LoS: Length of Stay; MICU: Medical Intensive Care Unit; NA: not applicable; NPs: nurse practitioners; MDs: medical doctors; PCPs: primary care providers; PAs: physician assistant/associates; QALY: Quality Adjusted Life Years; SBP: systolic blood pressure; USA: United States of America; WT: wait times.

Quality of care was assessed by patient outcomes, the process of care, accessibility of care, and the cost of care.

#### Patient outcomes

Regarding *Patient Outcome Evaluations*, data in 30 studies were assessed. In 13 studies, the care provided by a PA was the same as the physician’s usual care [16, 18, 20, 25, 26, 31, 32, 36, 41, 47, 52–54]. In 16 studies, the quality improved when the PA replaced a physician or was added as a member of a medical or surgical team [17, 21–23, 27, 28, 33, 34, 37, 38, 43, 46, 49, 51, 52, 54]. Two studies showed a mixed outcome; one improved outcome and one remained the same [46, 50]. Types of PA improvement varied from a reduction in complications of care [21–23, 28, 50], lower mortality [42], less hospitalization and readmissions [33, 38, 43, 51], fewer visits [37], and one demonstrated improvement in patient quality of life [27]. Patient satisfaction of PAs did not significantly differ from the patient satisfaction of a physician in the three studies that reported this outcome. However, patients did not always distinguish that the PA was not a physician [16, 25, 51].

#### Process of care

In five studies, the process of care remained the same [19, 25, 27, 31, 35], and in four studies, the outcome improved with the addition of a PA [28, 30, 32, 34]. Improvements were the use of thrombosis prophylaxis, beta-blockers, statins, or monitoring of blood pressure and blood glucose.

#### Provider outcomes

No studies reported the broader aspects of provider outcomes, such as workload or job satisfaction.

#### Accessibility of care

Four emergency department or acute care studies measured patient accessibility [15, 16, 28, 30]. Three studies reported a decreased waiting time [15, 16, 28], and two studies showed a reduction in the proportion of patients leaving without being seen [15, 30].

#### Costs of care

Twenty nine studies measured cost of care [14, 15, 18–20, 22, 23–30, 32,34, 35, 38, 39, 42, 46–54]. In 18 studies, the cost-effectiveness had been operationalized by the length of a hospital or inpatient stay (LoS), length of visit (LoV) or length of procedure time. In three studies the PAs led to an increase in LoS [29, 34, 53] and in three studies no difference was found in either LoV or LoS [26, 44, 50]. In 17 studies, the use of the PA led to a reduction in the overall cost of care [15, 19, 20, 22–24, 28–30, 32, 34, 35, 39, 42, 46, 52, 54].

The cost of care, in monetary terms, measured in 11 studies, decreased with the introduction of a PA, or the results were equal to that of a physician alone (whether as a physician replacement or to improve the process of care) [14, 24–27, 29, 35, 38, 39, 51, 53.

In one study, the cost of care by the PA was slightly greater than the physician’s care [53]. In another case, the PA provided a financial benefit when the reimbursement was at least 80% of an MD’s charge [47].

Two studies [20, 22] researched the procedural times in cardiac angioplasty between cardiology fellows and a cardiology PA. The PA produced slightly faster procedure times with less fluoroscopic exposure time.

For the most part, the reviewed studies in Table 2 did not produce a significant ‘differences of effect’ analysis. We note that in two ambulatory studies, the employment of the PA was associated with a slightly longer patient LoV (by a few hours). However, the cost of patient care when delivered by a physician exceeded the cost of care provided by PA [25, 29].

Three studies examined care outcomes by assessing cost-benefit and cost-utility—measuring the downstream cost-effectiveness of care or services [25, 38, 48]. In the Hooker 2002 study [48] and the Morgan 2019 study [38], the PAs did not negate their cost-benefit of less expensive labor by ordering more resources for an episode of care. In addition to the reduced labor cost, the medical resources used for an episode of care were less in the aggregate for the PA than the matched physician’s resources for the same episode of care.

In five studies, the PA was employed not as a direct replacement for a physician but in response to increased demand for care [14–18]. Still, when added to the medical staff, the PA significantly improved the throughput of patient services (e.g., maxillofacial surgery, emergency department, or lung procurement for transplantation). In each instance, the inclusion of a PA resulted in time per patient saved. When a PA was introduced in a newly created fast track system in the emergency department, the ‘through put’ of patients improved, and patient waiting time decreased [16]. In these studies, no calculation was made of cost-effectiveness in terms of hospital, training, or healthcare costs at a national level. Nor were there any studies that researched the provider’s workload or job satisfaction.

## Discussion

This review of 39 studies involved synthesizing the evidence on the cost-effectiveness of PA employment. Thirty-two studies presented a retrospective data analysis. The majority of the research focused on a physician substitution effect (34 out of 39 studies). Five studies focused on the impact of PA employment along with their contribution to the efficient production of medical services [14–18]. While the retrospective studies were methodological sound, such *ex post facto* design is of lower grade than prospective ones. At the same time, higher levels of evidence, such as randomized controlled trials, are not often applicable as it is challenging to randomize healthcare workers since patients cannot be blinded to healthcare professionals.

Throughout the assessed reports, the question raised most often was whether the PA provided adequate care, cost-efficient care, or improved quality of care. In the aggregate, the costs of care were improved in 24 studies. In 16 cases, the quality of care was the same as that provided by a physician, and only in two studies did the visit time attributed to the PA lengthen [27, 53]. In one study, the consult time of the PA slightly increased compared to the physician’s consult time [25].

Rarely did these studies examine the broader organizational effect of whether the addition of a provider improves overall organizational efficiency. Drennan *et al*. point out that when the PA’s service was incorporated in the cost-effectiveness analysis, this addition could have a broader impact on the cost of health services through referrals and prescriptions [25]. However, the authors concluded no significant differences in physician and PA rates of prescribing, ordering, referring, and consultation was found. As such, the costs were not assigned.

In terms of procedures, the outcome of circumcisions performed by a PA did not differ statistically from those of physicians. In contrast, the effects of performing surgical abortions, angioplasties, colonoscopies, and explanting lungs by PAs produced better outcomes compared with the physician’s performance.

As a result of this systematic review, it is apparent that PAs are cost-effective in their delivery of patient care. Furthermore, their role as team members improved the quality of care through the input, throughput, or output. Although the labor cost of a PA versus a physician was implied in 15 studies, it was only categorically addressed in the Grzybicki [47], Hooker [49], and Timmermans study [27]. Aside from these examples, the implication is that physician employment cost and educational costs are higher than a PA.

The findings that emerge from this consolidated analysis are generalizable. They transcend five countries and represent the broad span of PA employment; acute care settings, medical and surgical wards, proceduralists, and facilitators of patient throughput. As a timeline, the published dates of the studies represent almost half a century of critical observation of PAs (1977–2021). The included studies offer a timeframe of cost-effectiveness of emerging roles of PAs and how their use expanded from their early introduction in small practices to contemporary medical centers in the 21^st^ century.

The first economic studies using a time-motion method to observe the interaction of PAs and physicians regarding patient care were in the USA and published at a time when the development of the profession was still in its infancy [6, 14]. Early studies included some details of the PA, then known as a “new health professional,” and drew on the limited literature known at the time [57].

In studies before the 1980s, the PA often worked in a protocol-driven context [58]. In observations after the 1980s, the PA profession was more established in healthcare and similar to today’s professional profile, where the PA executes tasks independently. Their contemporary activity is viewed as an integrated member of a medical team [59].

From the 1990s onwards, the PA became more of a substitute for physician services in the role of a modern team player with a set of responsibilities [22, 29, 32, 38, 43, 47, 54]. By the new century, more countries had adopted the PA concept and drew on the American experience to develop their own professional PA profile [16, 23, 25, 31, 42].

Another observation of adding a PA was based on the decades of experience in the US and Canada for new PA adopters in Africa, Australia, and Europe. As the PA was considered in Europe in the new century, the implementers could draw on the experience, literature, demonstration studies, government reports, and observations of PAs at work to know how to best use their services and define their role [25]. By the second decade, the economics of their effectiveness had become more rigorous, as seen in the study of Timmerman and colleagues on cost-utility and Morgan’s and colleague’s studies of the cost-effectiveness of chronic disease management [38, 56]. In essence, each team of researchers was able to sophistically account for the downstream effect of PA utility on 17 inpatient wards across the Netherlands and 170 VA medical centers with their associated 1,400 outpatient clinics.

When the various research questions posed in the included studies are analyzed, the PA’s Scope of Practice (SoP) differed. Sometimes the PA’s SoP was narrow; for example, independently performed surgical procedures as in circumcisions, lungs harvesting, surgical abortions, and cardiac catheterization. In other situations, they had broad medical tasks backfilling the physician’s role on a ward or as an additional provider in an acute care setting with a commensurate SoP. In none of the articles did the researchers relate the SoP to the fourth goal in the “quadruple aim” of healthcare (i.e., taking care of health professionals) [60]. That raises the question of whether the performance of any procedure contributes to the experience of joy in their work as healthcare professionals [61]. However, the analysis of a half-century of PA job satisfaction literature suggests that almost all PAs find their role satisfying [62].

For the most part, the studies took place after the PA had been introduced into the organizational setting. In these situations, the outcomes before and after were compared. In five studies, the PA was added to a team [e.g., as part of a hospitalist service] or as a need to expand the medical staff [14–18]. Along with introducing a PA, organizational changes reflected on how services would be enhanced or improved. An example of organizational change is illustrated by Decloe et al. [42]. The PA was added to the infectious disease consulting service to mitigate the length of stay and patient morbidity and mortality in a Canadian hospital [42]. In another study, the medical residents that served as hospitalists were replaced with PA hospitalists in a small community hospital [43]. Both settings required significant organizational changes in staffing, hospital bylaws, on-boarding, and oversight of the PA.

We note that in the majority of studies in this review, the profiles of the PAs were missing. Most findings came up short on information as to the experience the PA brought to the setting. The exception was the de Lusignan study that noted the provider’s gender and experience [44].

Supervision of the PA by the physician was considered a necessary activity, especially during the first decade or so of the introduction of the PA profession. When a supervising physician took time off from their patient schedule to supervise the PA’s care or medical notes, the time was deducted from the PA’s employment benefits [6]. In 11 studies, this variable was noted, but only one study calculated the economic effect [27]. Many studies indicated that when comparing medical or surgical residents and PAs, the supervision by an attending physician or senior consultant was equal. Two studies identified that the use of the PA saved time for the medical specialist without having operationalized it further [18, 28].

Finally, we note that the effects of introducing a PA in several studies can be seen from the perspective of complex organizational change. The evaluation of a PA’s introduction, often as a new health professional in the chain of care, is not the same as a treatment intervention. One of the first scholars of PA effectiveness noted: *“As a theory*, *productivity is a simple concept*: *it measures changes in the total output that occurs when small changes are made in one factor of production*, *with all other factors and circumstances held constant*. *Because these conditions can be met in the real world only rarely*, *productivity numbers are almost always rough estimates*. *Certainly*, *that is the case concerning PAs*.*”* [6].

### Limitations

One limitation of this analysis is that the settings and the outcome parameters differed across studies, and the characteristics of the PA were often missing. More granular PA and physician information is needed to understand what could be influencing or confounding variables that affect the actual outcome. Variables missing across almost all studies are the experience, educational level, number of involved PAs, and their age, gender, and background.

Another limitation was the need to separate the outcomes of the employment of the PA and NP. We omitted studies where the combined labor was not isolated. In five cases, we inquired whether the two providers could be separated for analysis. Understanding where the division of labor exists when three medical professionals work together is a health services research area that needs further exploration.

One strength of this systematic review was the reliance on peer-reviewed and published studies. As a result, various government-initiated PA demonstration projects promulgated as reports were excluded as not peer-reviewed [referred to as ‘grey literature’]. Another strength was the breadth of the search that provided clear insight into the PA profession’s different effects and development. With the help of an experienced librarian, the research question was carefully operationalized. Combined with a reference check at the end of the process, the risk of missing relevant articles was significantly reduced.

## Conclusion

The PA of the 21^st^ century is a semi-autonomous health professional who is a part of contemporary medical treatment teams. When peer-reviewed published studies spanning three continents were examined for quality of care, accessibility, and cost-effectiveness of employment, the PA was comparable to the physician in producing similar results in almost every case. Although some of the studies suggest that the addition of a PA resulted in a similar quality of care as physicians, in a few instances, their utilization enhanced the overall quality of care. In most instances, the introduction of a PA leads to the same or an improved quality of care, and their employment is cost-efficient when considering the labor and educational costs. These economic findings were observed in prospective and retrospective designs and various settings, whether primary care in outpatient offices or secondary [hospital-based] care. The results of the collective studies have produced a sizeable contextual understanding of efficient outcomes of care when the PA is a part of the medical team.

## Supporting information

S1 Checklist (DOCX)

S1 Appendix (DOCX)

S2 Appendix (DOCX)

## Abbreviations

AF: Air Force
CA: Canada
CINAHL: Cumulative Index to Nursing and Allied Health Literature
ED: Emergency Department [includes A & E]
ICU: Intensive care units
IR: Ireland, Republic of
MD: physician/medical doctor. Includes American DOs
NL: Netherlands
NP: Nurse Practitioner
NS: Not stated in the manuscript
OEM: Occupational and Environmental Medicine
PA: Physician Assistant or Physician Associate
QALY: Quality Adjusted Life Year
ROBINS-I: Risk of Bias in Non-randomized Studies of Interventions
SA: South Africa
SoP: Scope of Practice
UK: United Kingdom
USA: United States of America
VA: Department of Veterans Affairs
VHA: Veterans Health Administration
WOS: Web of Science
