# Supplementary material for: The cost-effectiveness of physician assistants/associates: A systematic review of international evidence
Source: PLoS One. 2021 Nov 1;16(11):e0259183. doi: 10.1371/journal.pone.0259183 (PMC8559935; doi:10.1371/journal.pone.0259183)
Supplement: S1 Appendix — (DOCX) [file pone.0259183.s002.docx]

**Appendix 1:**

| ROBIN-I assessment | |  |  |  |  |  |  |  |  |
| --- | --- | --- | --- | --- | --- | --- | --- | --- | --- |
| **Author** | Domain 1: confounding | Domain 2: selection | Domain 3: classification of intervention | Domain 4: deviation from interventions | Domain 5: missing data | Domain 6: measurement of outcomes | Domain 7: selection of reported result | ROBINS-I overall |  |
| Althausen 2013 | 3 | 1 | 1 | 3 | 1 | 1 | 1 | 2 |  |
| Arnopolin 2001 | 3 | 2 | 3 | ni | ni | 1 | 1 | 3 |  |
| Capstack 2016 | 2 | 1 | 1 | 3 | 1 | 1 | 1 | 2 |  |
| Costa 2013 | 4 | 1 | 3 | ni | ni | ni | 1 | 4 |  |
| Decloe 2015 | 4 | 4 | 1 | 1 | ni | 1 | 1 | 2 |  |
| Dhuper 2009 | 4 | 2 | 1 | 2 | ni | 2 | 1 | 3 |  |
| Drennan 2015 | 2 | 1 | 1 | 1 | 2 | 1 | 1 | 2 |  |
| Everett 2019 | 1 | 1 | 1 | 1 | 1 | 1 | 1 | 1 |  |
| Fung 2020 | 1 | 1 | 1 | 1 | 1 | 1 | 1 | 1 |  |
| Glotzbecker 2012 | 2 | 1 | 2 | 1 | 1 | 1 | 1 | 1 |  |
| Goldman 2004 | 2 | 1 | 1 | 1 | 3 | 1 | 1 | 1 |  |
| Grzybicki 2002 | 1 | 1 | 1 | 1 | 1 | 1 | 1 | 1 |  |
| Hooker 2002 | 1 | 1 | 1 | 1 | 1 | 1 | 1 | 1 |  |
| Hooker 2004 | 1 | 1 | 1 | 1 | 1 | 1 | 1 | 1 |  |
| Kawar 2011 | 2 | 1 | 1 | 1 | 1 | 1 | 1 | 1 |  |
| Krasuki 2003 | 2 | 1 | 1 | 1 | 1 | 1 | 1 | 2 |  |
| Kuo 2013 | 1 | 1 | 1 | 1 | 1 | 1 | 1 | 1 |  |
| Morgan 2008 | 1 | 1 | 2 | 2 | 1 | 1 | 1 | 2 |  |
| Nestler 2012 | 1 | 1 | 1 | 1 | 1 | 1 | 1 | 1 |  |
| Ngcobo 2017 | 1 | 1 | 1 | 1 | 1 | 1 | 1 | 1 |  |
| Oswanski 2004 | 2 | 1 | 1 | 1 | 1 | 1 | 1 | 1 |  |
| Pavlik 2017 | 2 | 1 | 1 | 1 | 1 | 1 | 1 | 1 |  |
| Resnick 2016 | 3 | 1 | 1 | 1 | 3 | 1 | 1 | 2 |  |
| Singh 2011 | 3 | 1 | 1 | 1 | 1 | 1 | 1 | 2 |  |
| Theunissen 2014 | 1 | 1 | 1 | 1 | 1 | 1 | 1 | 1 |  |
| Timmermans 2017 | 1 | 1 | 1 | 3 | 1 | 1 | 1 | 1 |  |
| Tompkins 1977 | 1 | 1 | 1 | 1 | 1 | 3 | 1 | 1 |  |
| Van Rhee 2002 | 1 | 1 | 2 | 1 | 1 | 1 | 2 | 1 |  |
| Yang 2018 | 1 | 1 | 1 | 1 | 1 | 1 | 1 | 1 |  |
| Jackson 2018 | 1 | 1 | 1 | 1 | 1 | 1 | 1 | 1 |  |
| Faza 2018 | 1 | 1 | 1 | 1 | 1 | 1 | 1 | 1 |  |
| Roy 2008 | 2 | 1 | 1 | 1 | 1 | 1 | 1 | 1 |  |
| de la Roche 2021 | 1 | 1 | 1 | 1 | 3 | 3 | 1 | 2 |  |
| Malloy 2020 | 1 | 1 | 1 | 1 | 2 | 1 | 1 | 1 |  |
| DeMots 1987 | 3 | 1 | 1 | 1 | 3 | 3 | 1 | 3 |  |
| Morgan 2019 | 1 | 1 | 1 | 1 | 1 | 1 | 1 | 1 |  |
| Fejleh 2020 | 1 | 1 | 3 | 1 | 1 | 1 | 1 | 1 |  |
| Halter 2020 | 1 | 1 | 1 | 1 | 1 | 1 | 1 | 1 |  |
| Smith 2020 | 1 | 1 | 1 | 1 | 1 | 1 | 1 | 1 |  |
|  | 1 | 2 | 3 | 4 | 5 | 6 | 7 | 8 |  |
| 1 | 22 | 36 | 33 | 32 | 29 | 34 | 38 | 26 |  |
| 2 | 9 | 2 | 3 | 2 | 2 | 1 | 1 | 9 |  |
| 3 | 5 | 0 | 3 | 0 | 4 | 3 | 0 | 3 |  |
| 4 | 3 | 1 | 0 | 3 | 0 | 0 | 0 | 1 |  |
| 0 | 0 | 0 | 0 | 2 | 4 | 1 | 0 | 0 |  |
|  | 39 | 39 | 39 | 39 | 39 | 39 | 39 | 39 |  |
| Risk of bias assessment: 0 No information; 1 Low; 2 Moderate; 3 Serious; 4 Critical | | | | | | | | | |
| 0 [no information] was assessed as equivalent to “Serious” [3] | | | | | | | | | |
|  | | | | | | | | | |
