# Supplementary material for: The cost-effectiveness of physician assistants/associates: A systematic review of international evidence
Source: PLoS One. 2021 Nov 1;16(11):e0259183. doi: 10.1371/journal.pone.0259183 (PMC8559935; doi:10.1371/journal.pone.0259183)
Supplement: S2 Appendix — (DOCX) [file pone.0259183.s003.docx]

**S2 APPENDIX. SEARCH STRATEGY**

**Cinahl**

S1 MH "Physician Assistants" OR (TI ((physician N1 (assistant* OR associate* OR extender* OR substitute*)) OR (medical W1 extender*) OR (advanced W1 provider*) OR (emergency W1 practitioner*) OR ((Midlevel OR (mid W1 level) OR ((non OR none) W1 physician*) OR nonphysician*) W2 (clinic* OR ((health OR healthcare) W2 (professional* OR provider* OR worker*)) OR personnel OR practitioner* OR professional* OR provider* OR staff OR worker* OR specialist* OR (first W1 assistant*))) OR (midlevel W1 health W1 care) OR (advance* W1 practice W1 provider*))) OR (AB ((physician N1 (assistant* OR associate* OR extender* OR substitute*)) OR (medical W1 extender*) OR (advanced W1 provider*) OR (emergency W1 practitioner*) OR ((Midlevel OR (mid W1 level) OR ((non OR none) W1 physician*) OR nonphysician*) W2 (clinic* OR ((health OR healthcare) W2 (professional* OR provider* OR worker*)) OR personnel OR practitioner* OR professional* OR provider* OR staff OR worker* OR specialist* OR (first W1 assistant*))) OR (midlevel W1 health W1 care) OR (advance* W1 practice W1 provider*))) OR (SU ((physician N1 (assistant* OR associate* OR extender* OR substitute*)) OR (medical W1 extender*) OR (advanced W1 provider*) OR (emergency W1 practitioner*) OR ((Midlevel OR (mid W1 level) OR ((non OR none) W1 physician*) OR nonphysician*) W2 (clinic* OR ((health OR healthcare) W2 (professional* OR provider* OR worker*)) OR personnel OR practitioner* OR professional* OR provider* OR staff OR worker* OR specialist* OR (first W1 assistant*))) OR (midlevel W1 health W1 care) OR (advance* W1 practice W1 provider*)))

S2 (MH "Costs and Cost Analysis+" OR MH "Economic Aspects of Illness" OR MH "Insurance+" OR MH "Referral and Consultation+") OR (TI (((Spend* OR Fund* OR Expen* OR Budget) N1 (control* OR saving* OR care OR health* OR high OR medical)) OR champus OR (Claim* N1 (analysis OR review* OR Analysis)) OR Coinsurance* OR (Competitive N1 (Health* OR medical) N1 Plan*) OR Costeffect* OR Deductible* OR (direct W1 cost*) OR (Economic N1 evaluat*) OR (Health N1 Benefit N1 Plan*) OR insuran* OR (managed N1 car*) OR medicare OR (Preferred N1 provider*) OR Reimburs* OR (Third N1 Party N1 Pay*) OR (Usage N1 reduction*) OR (Value N1 Based N1 Purchas*) OR (Worker* N1 Compensation*) OR (Return N1 on N1 investment*) OR ROI OR ((Cost OR costs) N1 (allocat* OR analy* OR apportionment* OR benefit* OR compar* OR contain* OR control* OR decreas* OR effective* OR Efficien* OR evaluat* OR increase* OR manag* OR minimi* OR reduc* OR saving* OR sharing OR shifting* OR minimization OR minimization OR (health N1 care) OR health* OR high* OR low* OR increas* OR medical OR rising OR societal OR Treatment)))) OR (AB (((Spend* OR Fund* OR Expen* OR Budget) N1 (control* OR saving* OR care OR health* OR high OR medical)) OR champus OR (Claim* N1 (analysis OR review* OR Analysis)) OR Coinsurance* OR (Competitive N1 (Health* OR medical) N1 Plan*) OR Costeffect* OR Deductible* OR (direct W1 cost*) OR (Economic N1 evaluat*) OR (Health N1 Benefit N1 Plan*) OR insuran* OR (managed N1 car*) OR medicare OR (Preferred N1 provider*) OR Reimburs* OR (Third N1 Party N1 Pay*) OR (Usage N1 reduction*) OR (Value N1 Based N1 Purchas*) OR (Worker* N1 Compensation*) OR (Return N1 on N1 investment*) OR ROI OR ((Cost OR costs) N1 (allocat* OR analy* OR apportionment* OR benefit* OR compar* OR contain* OR control* OR decreas* OR effective* OR Efficien* OR evaluat* OR increase* OR manag* OR minimi* OR reduc* OR saving* OR sharing OR shifting* OR minimization OR minimization OR (health N1 care) OR health* OR high* OR low* OR increas* OR medical OR rising OR societal OR Treatment)))) OR (SU (((Spend* OR Fund* OR Expen* OR Budget) N1 (control* OR saving* OR care OR health* OR high OR medical)) OR champus OR (Claim* N1 (analysis OR review* OR Analysis)) OR Coinsurance* OR (Competitive N1 (Health* OR medical) N1 Plan*) OR Costeffect* OR Deductible* OR (direct W1 cost*) OR (Economic N1 evaluat*) OR (Health N1 Benefit N1 Plan*) OR insuran* OR (managed N1 car*) OR medicare OR (Preferred N1 provider*) OR Reimburs* OR (Third N1 Party N1 Pay*) OR (Usage N1 reduction*) OR (Value N1 Based N1 Purchas*) OR (Worker* N1 Compensation*) OR (Return N1 on N1 investment*) OR ROI OR ((Cost OR costs) N1 (allocat* OR analy* OR apportionment* OR benefit* OR compar* OR contain* OR control* OR decreas* OR effective* OR Efficien* OR evaluat* OR increase* OR manag* OR minimi* OR reduc* OR saving* OR sharing OR shifting* OR minimization OR minimization OR (health N1 care) OR health* OR high* OR low* OR increas* OR medical OR rising OR societal OR Treatment))))

S3 (MH "Waiting Lists") OR (TI (waitlist* OR delist* OR (wait*N1 (period OR list* OR time)))) OR (AB (waitlist* OR delist* OR (wait*N1 (period OR list* OR time)))) OR (SU (waitlist* OR delist* OR (wait*N1 (period OR list* OR time))))

S4 (MH "Hospitalization") OR (MH "Diagnosis-Related Groups") OR (MH "Outliers, DRG") OR (MH "Transfer, Intrahospital") OR (MH "Length of Stay") OR (MH "Patient Admission") OR (MH "Patient Discharge+") OR (MH "Patient Dumping") OR (MH "Readmission") OR (TI (Hospitaliz* OR Hospitalis* OR Admission* OR Discharg* OR Handoff* OR (Hand* W1 (Over* OR off)) OR (Sign W1 Out*) OR Signout* OR Handover* OR Readmis* OR ((healthcare OR care OR patient) N1 (transfer* OR transition*)) OR (Patient N1 (Turfing* OR Dumping*)) OR ((Duration OR Hospital OR Length*) N1 stay*))) OR (AB (Hospitaliz* OR Hospitalis* OR Admission* OR Discharg* OR Handoff* OR (Hand* W1 (Over* OR off)) OR (Sign W1 Out*) OR Signout* OR Handover* OR Readmis* OR ((healthcare OR care OR patient) N1 (transfer* OR transition*)) OR (Patient N1 (Turfing* OR Dumping*)) OR ((Duration OR Hospital OR Length*) N1 stay*))) OR (SU (Hospitaliz* OR Hospitalis* OR Admission* OR Discharg* OR Handoff* OR (Hand* W1 (Over* OR off)) OR (Sign W1 Out*) OR Signout* OR Handover* OR Readmis* OR ((healthcare OR care OR patient) N1 (transfer* OR transition*)) OR (Patient N1 (Turfing* OR Dumping*)) OR ((Duration OR Hospital OR Length*) N1 stay*)))

S5 (MH "Mortality+") OR MW "MO" OR (TI (Mortalit* OR (Case N1 Fatality N1 Rate*) OR (Death N1 Rate*) OR (Fatal N1 Outcome*))) OR (AB (Mortalit* OR (Case N1 Fatality N1 Rate*) OR (Death N1 Rate*) OR (Fatal N1 Outcome*))) OR (SU (Mortalit* OR (Case N1 Fatality N1 Rate*) OR (Death N1 Rate*) OR (Fatal N1 Outcome*)))

S6 (MH "Morbidity+") OR (TI (morbidit* OR comorbidit*)) OR (AB (morbidit* OR comorbidit*)) OR (SU (morbidit* OR comorbidit*))

S7 (MH "Quality of Life+") OR (MH "Health Status+") OR (MH "Activities of Daily Living+") OR (MH "Quality-Adjusted Life Years") OR (TI ((Quality N1 Adjusted N1 Years) OR QALY OR (Healthy N1 Year* N1 Equivalent*) OR (Adjusted N1 Life N1 Year*) OR (life N1 qualit*) OR (quality N1 of N1 life) OR (health N1 status) OR (level N1 of N1 health) OR (health N1 level*) OR qol OR hrql OR hrqol OR (activities N1 of N1 daily N1 living) OR (daily N1 activit*) OR adl OR (chronic N1 limitation N1 of N1 activit*))) OR (AB ((Quality N1 Adjusted N1 Years) OR QALY OR (Healthy N1 Year* N1 Equivalent*) OR (Adjusted N1 Life N1 Year*) OR (life N1 qualit*) OR (quality N1 of N1 life) OR (health N1 status) OR (level N1 of N1 health) OR (health N1 level*) OR qol OR hrql OR hrqol OR (activities N1 of N1 daily N1 living) OR (daily N1 activit*) OR adl OR (chronic N1 limitation N1 of N1 activit*))) OR (SU ((Quality N1 Adjusted N1 Years) OR QALY OR (Healthy N1 Year* N1 Equivalent*) OR (Adjusted N1 Life N1 Year*) OR (life N1 qualit*) OR (quality N1 of N1 life) OR (health N1 status) OR (level N1 of N1 health) OR (health N1 level*) OR qol OR hrql OR hrqol OR (activities N1 of N1 daily N1 living) OR (daily N1 activit*) OR adl OR (chronic N1 limitation N1 of N1 activit*)))

S8 (MH "Patient Satisfaction") OR (TI (Patient N1 (Satisfaction* OR preference* OR experienc*))) OR (AB (Patient N1 (Satisfaction* OR preference* OR experienc*))) OR (SU (Patient N1 (Satisfaction* OR preference* OR experienc*)))

S9 (MH "Patient Compliance+") OR (TI ((medication OR Therapeutic OR Treatment OR Patient) N1 (Adher* OR Cooperat* OR ((Non OR none) N1 (Compli* OR adher*)) OR Nonadher* OR Noncompli*))) OR (AB ((medication OR Therapeutic OR Treatment OR Patient) N1 (Adher* OR Cooperat* OR ((Non OR none) N1 (Compli* OR adher*)) OR Nonadher* OR Noncompli*))) OR (SU ((medication OR Therapeutic OR Treatment OR Patient) N1 (Adher* OR Cooperat* OR ((Non OR none) N1 (Compli* OR adher*)) OR Nonadher* OR Noncompli*)))

S10 (MH "Patient Safety") OR (MH "Adverse Health Care Event+") OR (MH "Health Care Errors+") OR (MH "Sentinel Event") OR (MH "Fire Safety") OR (MH "Electrical Safety") OR (MH "Chemical Safety") OR (MH "Radiation Safety") OR (TI (((Wrong N1 Procedure) OR Surgical OR Medical OR Diagnostic) N1 (Mistake* OR Error* OR (Wrong N1 Site N1 Surger*)) OR (Critical N1 Medical N1 Incident*) OR (Never N1 Event*) OR Misdiagnos* OR (False N1 (Negative OR positive) N1 Reaction*) OR ((Intraobserver OR Interobserver OR Observer*) N1 (Variation* OR bias OR Variabilit*)) OR (Near N1 Miss*) OR (Close N1 Call*) OR (Radiotherapy N1 Setup N1 Error*) OR ((Medication OR (Drug N1 Use)) N1 Error*) OR (patient N1 safet*))) OR (AB (((Wrong N1 Procedure) OR Surgical OR Medical OR Diagnostic) N1 (Mistake* OR Error* OR (Wrong N1 Site N1 Surger*)) OR (Critical N1 Medical N1 Incident*) OR (Never N1 Event*) OR Misdiagnos* OR (False N1 (Negative OR positive) N1 Reaction*) OR ((Intraobserver OR Interobserver OR Observer*) N1 (Variation* OR bias OR Variabilit*)) OR (Near N1 Miss*) OR (Close N1 Call*) OR (Radiotherapy N1 Setup N1 Error*) OR ((Medication OR (Drug N1 Use)) N1 Error*) OR (patient N1 safet*))) OR (SU (((Wrong N1 Procedure) OR Surgical OR Medical OR Diagnostic) N1 (Mistake* OR Error* OR (Wrong N1 Site N1 Surger*)) OR (Critical N1 Medical N1 Incident*) OR (Never N1 Event*) OR Misdiagnos* OR (False N1 (Negative OR positive) N1 Reaction*) OR ((Intraobserver OR Interobserver OR Observer*) N1 (Variation* OR bias OR Variabilit*)) OR (Near N1 Miss*) OR (Close N1 Call*) OR (Radiotherapy N1 Setup N1 Error*) OR ((Medication OR (Drug N1 Use)) N1 Error*) OR (patient N1 safet*)))

S11 (MH "Quality of Health Care") OR (MH "Program Evaluation") OR (TI (((care OR healthcare OR (Health N1 Care)) N1 Qualit*) OR ((Institutional OR Protocol OR Policy OR Guideline) N1 (adherence OR Compliance)) OR “Outcome and Process Assessment” OR (Structure N1 Process N1 Outcome N1 Triad*) OR (Donabedian N1 (Model* OR Triad*)) OR (Outcome* N1 (Assessment* OR Research* OR Stud* OR Measure*)) OR (Failure* N1 to N1 Rescue*) OR (Patient N1 Outcome* N1 Assessment*) OR (Patient N1 Centered N1 Outcome* N1 Research) OR (((Patient N1 Relevant) OR Rehabilitation OR Treatment OR (Patient N1 Reported) OR (Critical N1 Care)) N1 Outcome*) OR ((Treatment OR Clinical) N1 (Effectiveness* OR Efficac*)) OR (Treatment N1 Failure*) OR (Process N1 (Assessment* OR Measure*)) OR (((Professional N1 Review) OR (Peer N1 Review) OR (Professional N1 Standards N1 Review)) N1 Organization*) OR PSRO OR “Utilization and Quality Control Peer Review Organizations” OR (Program N1 (Evaluation* OR Sustainabilit* OR Effectiveness OR Appropriateness)) OR (Best N1 Practice N1 Analysis) OR Benchmark* OR (((Health N1 Care N1 Quality) OR (Healthcare N1 Quality)) N1 (Assurance* OR Assessment*)) OR (Alert N1 Fatigue N1 Health N1 Personnel) OR (Laboratory N1 Proficiency N1 Test*) OR (Near N1 Miss*) OR (Close N1 Call*) OR (PIM N1 List*) OR (Potentially N1 Inappropriate N1 Medication*) OR (Beers N1 Criteria*) OR (Beers N1 Potentially N1 Inappropriate N1 Medication*) OR STOPP OR (Screening N1 Tool N1 of N1 Older N1 Person* N1 Potentially N1 Inappropriate N1 Prescription*))) OR (AB (((care OR healthcare OR (Health N1 Care)) N1 Qualit*) OR ((Institutional OR Protocol OR Policy OR Guideline) N1 (adherence OR Compliance)) OR “Outcome and Process Assessment” OR (Structure N1 Process N1 Outcome N1 Triad*) OR (Donabedian N1 (Model* OR Triad*)) OR (Outcome* N1 (Assessment* OR Research* OR Stud* OR Measure*)) OR (Failure* N1 to N1 Rescue*) OR (Patient N1 Outcome* N1 Assessment*) OR (Patient N1 Centered N1 Outcome* N1 Research) OR (((Patient N1 Relevant) OR Rehabilitation OR Treatment OR (Patient N1 Reported) OR (Critical N1 Care)) N1 Outcome*) OR ((Treatment OR Clinical) N1 (Effectiveness* OR Efficac*)) OR (Treatment N1 Failure*) OR (Process N1 (Assessment* OR Measure*)) OR (((Professional N1 Review) OR (Peer N1 Review) OR (Professional N1 Standards N1 Review)) N1 Organization*) OR PSRO OR “Utilization and Quality Control Peer Review Organizations” OR (Program N1 (Evaluation* OR Sustainabilit* OR Effectiveness OR Appropriateness)) OR (Best N1 Practice N1 Analysis) OR Benchmark* OR (((Health N1 Care N1 Quality) OR (Healthcare N1 Quality)) N1 (Assurance* OR Assessment*)) OR (Alert N1 Fatigue N1 Health N1 Personnel) OR (Laboratory N1 Proficiency N1 Test*) OR (Near N1 Miss*) OR (Close N1 Call*) OR (PIM N1 List*) OR (Potentially N1 Inappropriate N1 Medication*) OR (Beers N1 Criteria*) OR (Beers N1 Potentially N1 Inappropriate N1 Medication*) OR STOPP OR (Screening N1 Tool N1 of N1 Older N1 Person* N1 Potentially N1 Inappropriate N1 Prescription*))) OR (SU (((care OR healthcare OR (Health N1 Care)) N1 Qualit*) OR ((Institutional OR Protocol OR Policy OR Guideline) N1 (adherence OR Compliance)) OR “Outcome and Process Assessment” OR (Structure N1 Process N1 Outcome N1 Triad*) OR (Donabedian N1 (Model* OR Triad*)) OR (Outcome* N1 (Assessment* OR Research* OR Stud* OR Measure*)) OR (Failure* N1 to N1 Rescue*) OR (Patient N1 Outcome* N1 Assessment*) OR (Patient N1 Centered N1 Outcome* N1 Research) OR (((Patient N1 Relevant) OR Rehabilitation OR Treatment OR (Patient N1 Reported) OR (Critical N1 Care)) N1 Outcome*) OR ((Treatment OR Clinical) N1 (Effectiveness* OR Efficac*)) OR (Treatment N1 Failure*) OR (Process N1 (Assessment* OR Measure*)) OR (((Professional N1 Review) OR (Peer N1 Review) OR (Professional N1 Standards N1 Review)) N1 Organization*) OR PSRO OR “Utilization and Quality Control Peer Review Organizations” OR (Program N1 (Evaluation* OR Sustainabilit* OR Effectiveness OR Appropriateness)) OR (Best N1 Practice N1 Analysis) OR Benchmark* OR (((Health N1 Care N1 Quality) OR (Healthcare N1 Quality)) N1 (Assurance* OR Assessment*)) OR (Alert N1 Fatigue N1 Health N1 Personnel) OR (Laboratory N1 Proficiency N1 Test*) OR (Near N1 Miss*) OR (Close N1 Call*) OR (PIM N1 List*) OR (Potentially N1 Inappropriate N1 Medication*) OR (Beers N1 Criteria*) OR (Beers N1 Potentially N1 Inappropriate N1 Medication*) OR STOPP OR (Screening N1 Tool N1 of N1 Older N1 Person* N1 Potentially N1 Inappropriate N1 Prescription*)))

S12 (MH "Workload") OR (MH "Task Performance and Analysis+") OR (TI ((Task N1 Performance*) OR (Critical N1 Incident N1 Techni*) OR workload* OR (work N1 load*))) OR (AB ((Task N1 Performance*) OR (Critical N1 Incident N1 Techni*) OR workload* OR (work N1 load*))) OR (SU ((Task N1 Performance*) OR (Critical N1 Incident N1 Techni*) OR workload* OR (work N1 load*)))

S13 (MH "Job Satisfaction+") OR (TI ((Job OR work) N1 Satisfaction)) OR (AB ((Job OR work) N1 Satisfaction)) OR (SU ((Job OR work) N1 Satisfaction))

S14 (MH "Organizational Efficiency+") OR (TI (efficien*OR inefficien* OR (clinical W1 effective*) OR productiv* OR effective* OR ineffective*)) OR (AB (efficien*OR inefficien* OR (clinical W1 effective*) OR productiv*)) OR (SU (efficien*OR inefficien* OR (clinical W1 effective*) OR productiv*))

S15 S2 OR S3 OR S4 OR S5 OR S6 OR S7 OR S8 OR S9 OR S10 OR S11 OR S12 OR S13 OR S14

S16 (MH "Clinical Trials+") OR (PT (Clinical trial)) OR (MH "Random Assignment") OR (MH "Quantitative Studies") OR (TX ((clini* N1 trial*) OR ((waitlist* OR (wait* and list*)) and (control* OR group)) OR "treatment as usual" OR tau OR (control* N3 (trial* OR study OR studies OR group*)) OR randomized OR randomised))

S17 (MH "Prospective Studies+") OR (MH "Double-Blind Studies") OR (MH "Single-Blind Studies") OR (MH "Triple-Blind Studies") OR (TX (cohort* OR ((Concurrent OR Incidence OR Followup* OR Prospective OR Longitudinal OR Retrospective OR (Follow N1 up*)) N1 (Study OR studies)) OR (Longitudinal N1 Surve*) OR (before N1 after)))

S18 (TX ((multi N1 (center* OR centre*) N1 stud*) OR (multicenter* N1 stud*)))

S19 S16 OR S17 OR S18

S20 S1 AND S15 AND S19

Results: 1,170

**Cochrane**

#1 ((physician NEAR/1 (assistant* OR associate* OR extender* OR substitute*)) OR (medical NEAR/1 extender*) OR (advanced NEAR/1 provider*) OR (emergency NEAR/1 practitioner*) OR ((Midlevel OR (mid NEAR/1 level) OR ((non OR none) NEAR/1 physician*) OR nonphysician*) NEAR/2 (clinic* OR ((health OR healthcare) NEAR/2 (professional* OR provider* OR worker*)) OR personnel OR practitioner* OR professional* OR provider* OR staff OR worker* OR specialist* OR (first NEAR/1 assistant*))) OR (midlevel NEAR/1 health NEAR/1 care) OR (advance* NEAR/1 practice NEAR/1 provider*)):ti,ab,kw

#2 (((Spend* OR Fund* OR Expen* OR Budget*) NEAR/1 (control* OR saving* OR care OR health* OR high OR medical)) OR champus OR (Claim* NEAR/1 (analysis OR review*)) OR Coinsurance* OR (Competitive NEAR/1 (Health* OR medical) NEAR/1 Plan*) OR Costeffect* OR Deductible* OR (direct NEAR/1 cost*) OR (Economic NEAR/1 evaluat*) OR (Health NEAR/1 Benefit* NEAR/1 Plan*) OR insuran* OR (managed NEAR/1 car*) OR medicare OR (Preferred NEAR/1 provider*) OR Reimburs* OR (Third NEAR/1 Party NEAR/1 Pay*) OR (Usage NEAR/1 reduction*) OR (Value NEAR/1 Based NEAR/1 Purchas*) OR (Worker* NEAR/1 Compensation*) OR (Return NEAR/1 on NEAR/1 investment*) OR ROI OR ((Cost OR costs) NEAR/1 (allocat* OR analy* OR apportionment* OR benefit* OR compar* OR contain* OR control* OR decreas* OR effective* OR Efficien* OR evaluat* OR increase* OR manag* OR minimi* OR reduc* OR saving* OR sharing OR shifting* OR minimization OR minimisation OR (health NEAR/1 care) OR health* OR high* OR low* OR increas* OR medical OR rising OR societal OR Treatment))):ti,ab,kw

#3 (waitlist* OR delist* OR (wait* NEAR/1 (period OR list* OR time))):ti,ab,kw

#4 (Hospitaliz* OR Hospitalis* OR Admission* OR Discharg* OR Handoff* OR (Hand* NEAR/1 (Over* OR off)) OR (Sign NEAR/1 Out*) OR Signout* OR Handover* OR Readmis* OR ((healthcare OR care OR patient*) NEAR/1 (transfer* OR transition*)) OR (Patient* NEAR/1 (Turfing* OR Dumping*)) OR ((Duration OR Hospital OR Length*) NEAR/1 stay*)):ti,ab,kw

#5 (Mortalit* OR (Case NEAR/1 Fatality NEAR/1 Rate*) OR (Death NEAR/1 Rate*) OR (Fatal NEAR/1 Outcome*)):ti,ab,kw

#6 (morbidit* OR comorbidit*):ti,ab,kw

#7 ((Quality NEAR/1 Adjusted NEAR/1 Years) OR QALY OR (Healthy NEAR/1 Year* NEAR/1 Equivalent*) OR (Adjusted NEAR/1 Life NEAR/1 Year*) OR (life NEAR/1 qualit*) OR (quality NEAR/1 of NEAR/1 life) OR (health NEAR/1 status) OR (level NEAR/1 of NEAR/1 health) OR (health NEAR/1 level*) OR qol OR hrql OR hrqol OR (activit* NEAR/1 of NEAR/1 daily NEAR/1 living) OR (daily NEAR/1 activit*) OR adl OR (chronic NEAR/1 limitation NEAR/1 of NEAR/1 activit*)):ti,ab,kw

#8 (Patient* NEAR/1 (Satisfaction* OR preference* OR experienc*)):ti,ab,kw

#9 ((medication OR Therapeutic OR Treatment OR Patient*) NEAR/1 (Adher* OR Cooperat* OR ((Non OR none) NEAR/1 (Compli* OR adher*)) OR Nonadher* OR Noncompli*)):ti,ab,kw

#10 ((Wrong NEAR/1 Procedure*) OR (Wrong NEAR/1 Site NEAR/1 Surger*) OR ((Surgical OR Medical OR Diagnostic) NEAR/1 (Mistake* OR Error*)) OR (Critical NEAR/1 Medical NEAR/1 Incident*) OR (Never NEAR/1 Event*) OR Misdiagnos* OR (False NEAR/1 (Negative OR positive) NEAR/1 Reaction*) OR ((Intraobserver OR Interobserver OR Observer*) NEAR/1 (Variation* OR bias OR Variabilit*)) OR (“Near” NEAR/1 Miss*) OR (Close NEAR/1 Call*) OR (Radiotherapy NEAR/1 Setup NEAR/1 Error*) OR ((Medication OR (Drug NEAR/1 Use)) NEAR/1 Error*) OR (patient* NEAR/1 safet*)):ti,ab,kw

#11 (((care OR healthcare OR (Health NEAR/1 Care)) NEAR/1 Qualit*) OR ((Institutional OR Protocol OR Policy OR Guideline) NEAR/1 (adherence OR Compliance)) OR “Outcome and Process Assessment” OR (Structure NEAR/1 Process NEAR/1 Outcome NEAR/1 Triad*) OR (Donabedian NEAR/1 (Model* OR Triad*)) OR (Outcome* NEAR/1 (Assessment* OR Research* OR Stud* OR Measure*)) OR (Failure* NEAR/1 to NEAR/1 Rescue*) OR (Patient NEAR/1 Outcome* NEAR/1 Assessment*) OR (Patient NEAR/1 (Centered OR centred) NEAR/1 Outcome* NEAR/1 Research) OR (((Patient NEAR/1 Relevant) OR Rehabilitation OR Treatment OR (Patient NEAR/1 Reported) OR (Critical NEAR/1 Care)) NEAR/1 Outcome*) OR ((Treatment OR Clinical) NEAR/1 (Effectiveness* OR Efficac*)) OR (Treatment NEAR/1 Failure*) OR (Process NEAR/1 (Assessment* OR Measure*)) OR (((Professional NEAR/1 Review*) OR (Peer NEAR/1 Review*) OR (Professional NEAR/1 Standards NEAR/1 Review*)) NEAR/1 Organization*) OR PSRO OR “Utilization and Quality Control Peer Review Organizations” OR (Program* NEAR/1 (Evaluation* OR Sustainabilit* OR Effectiveness OR Appropriateness)) OR (Best NEAR/1 Practice NEAR/1 Analysis) OR Benchmark* OR (((Health NEAR/1 Care NEAR/1 Quality) OR (Healthcare NEAR/1 Quality)) NEAR/1 (Assurance* OR Assessment*)) OR (Alert NEAR/1 Fatigue NEAR/1 Health NEAR/1 Personnel) OR (Laboratory NEAR/1 Proficiency NEAR/1 Test*) OR (Close NEAR/1 Call*) OR (PIM NEAR/1 List*) OR (Potentially NEAR/1 Inappropriate NEAR/1 Medication*) OR (Beers NEAR/1 Criteria*) OR (Beers NEAR/1 Potentially NEAR/1 Inappropriate NEAR/1 Medication*) OR STOPP OR (Screening NEAR/1 Tool NEAR/1 of NEAR/1 Older NEAR/1 Person* NEAR/1 Potentially NEAR/1 Inappropriate NEAR/1 Prescription*)):ti,ab,kw

#12 ((Task NEAR/1 Performance*) OR (Critical NEAR/1 Incident* NEAR/1 Techni*) OR workload* OR (work NEAR/1 load*)):ti,ab,kw

#13 ((Job OR work) NEAR/1 Satisfaction):ti,ab,kw

#14 (effective* OR ineffective*):ti

#15 (efficien*OR inefficien* OR (clinical NEAR/1 effective*) OR productiv*):ti,ab,kw

#16 #2 OR #3 OR #4 OR #5 OR #6 OR #7 OR #8 OR #9 OR #10 OR #11 OR #12 OR #13 OR #14 OR #15

#17 ((clini* NEAR/1 trial*) OR ((waitlist* OR (wait* and list*)) and (control* OR group)) OR "treatment as usual" OR tau OR (control* NEAR/3 (trial* OR study OR studies OR group*)) OR randomized OR randomised)

#18 (cohort* OR ((Concurrent OR Incidence OR Followup* OR Prospective OR Longitudinal OR Retrospective OR (Follow NEAR/1 up*)) NEAR/1 (Study OR studies)) OR (Longitudinal NEAR/1 Surve*) OR (before NEAR/1 after))

#19 ((multi NEAR/1 (center* OR centre*) NEAR/1 stud*) OR (multicenter* NEAR/1 stud*))

#20 #17 OR #18 OR #19

#21 #1 AND #16 AND #20

Results: 414

**Embase**

1 physician assistant/ or ((physician adj1 (associate or associates or extender* or substitute*)) or "physician assistant" or "physician assistants" or (advance* adj1 provider*) or (emergency adj1 practitioner*) or ((Midlevel or (mid adj1 level) or ((non or none) adj1 physician*) or nonphysician*) adj2 (clinician* or professional* or provider* or worker* or personnel or practitioner* or professional* or provider* or staff or worker* or specialist* or (first adj1 assistant*))) or (advance* adj1 practice adj1 provider*)).ti,ab,kw.

2 health economics/ or exp economic evaluation/ or exp "health care cost"/ or exp health insurance/ OR patient referral/ OR (((Spend* OR Fund* OR Expen* OR Budget*) ADJ1 (control* OR saving* OR care OR health* OR high OR medical)) OR champus OR (Claim* ADJ1 (analysis OR review*)) OR Coinsurance* OR (Competitive ADJ1 (Health* OR medical) ADJ1 Plan*) OR Costeffect* OR Deductible* OR (direct ADJ1 cost*) OR (Economic ADJ1 evaluat*) OR (Health ADJ1 Benefit* ADJ1 Plan*) OR insuran* OR (managed ADJ1 car*) OR medicare OR (Preferred ADJ1 provider*) OR Reimburs* OR (Third ADJ1 Party ADJ1 Pay*) OR (Usage ADJ1 reduction*) OR (Value ADJ1 Based ADJ1 Purchas*) OR (Worker* ADJ1 Compensation*) OR (Return ADJ1 on ADJ1 investment*) OR ROI OR ((Cost OR costs) ADJ1 (allocat* OR analy* OR apportionment* OR benefit* OR compar* OR contain* OR control* OR decreas* OR effective* OR Efficien* OR evaluat* OR increase* OR manag* OR minimi* OR reduc* OR saving* OR sharing OR shifting* OR minimization OR minimisation OR (health ADJ1 care) OR health* OR high* OR low* OR increas* OR medical OR rising OR societal OR Treatment))).ti,ab,kw.

3 (waitlist* OR delist* OR (wait* ADJ1 (period OR list* OR time))).ti,ab,kw.

4 hospitalization/ OR Diagnosis Related Group/ OR length of stay/ OR patient dumping/ OR hospital readmission/ OR (Hospitaliz* OR Hospitalis* OR Admission* OR Discharg* OR Handoff* OR (Hand* ADJ1 (Over* OR off)) OR (Sign ADJ1 Out*) OR Signout* OR Handover* OR Readmis* OR ((healthcare OR care OR patient*) ADJ1 (transfer* OR transition*)) OR (Patient* ADJ1 (Turfing* OR Dumping*)) OR ((Duration OR Hospital OR Length*) ADJ1 stay*)).ti,ab,kw.

5 exp mortality/ OR (Mortalit* OR (Case ADJ1 Fatality ADJ1 Rate*) OR (Death ADJ1 Rate*) OR (Fatal ADJ1 Outcome*)).ti,ab,kw.

6 morbidity/ OR (morbidit* OR comorbidit*).ti,ab,kw.

7 exp quality of life/ OR exp health status/ OR exp activity of daily living assessment/ OR ((Quality ADJ1 Adjusted ADJ1 Years) OR QALY OR (Healthy ADJ1 Year* ADJ1 Equivalent*) OR (Adjusted ADJ1 Life ADJ1 Year*) OR (life ADJ1 qualit*) OR (quality ADJ1 of ADJ1 life) OR (health ADJ1 status) OR (level ADJ1 of ADJ1 health) OR (health ADJ1 level*) OR qol OR hrql OR hrqol OR (activit* ADJ1 of ADJ1 daily ADJ1 living) OR (daily ADJ1 activit*) OR adl OR (chronic ADJ1 limitation ADJ1 of ADJ1 activit*)).ti,ab,kw.

8 patient satisfaction/ OR (Patient* ADJ1 (Satisfaction* OR preference* OR experienc*)).ti,ab,kw.

9 exp Patient Compliance/ OR ((medication OR Therapeutic OR Treatment OR Patient*) ADJ1 (Adher* OR Cooperat* OR ((Non OR none) ADJ1 (Compli* OR adher*)) OR Nonadher* OR Noncompli*)).ti,ab,kw.

10 exp patient safety/ or sentinel event/ or ((Wrong adj1 Procedure*) or (Wrong adj1 Site adj1 Surger*) or ((Surgical or Medical or Diagnostic) adj1 (Mistake* or Error*)) or (Critical adj1 Medical adj1 Incident*) or (Never adj1 Event*) or Misdiagnos* or (False adj1 (Negative or positive) adj1 Reaction*) or ((Intraobserver or Interobserver or Observer*) adj1 (Variation* or bias or Variabilit*)) or (Near adj1 Miss*) or (Close adj1 Call*) or (Radiotherapy adj1 Setup adj1 Error*) or ((Medication or (Drug adj1 "use")) adj1 Error*) or (patient* adj1 safet*)).ti,ab,kw.

11 exp Health Care quality/ OR (((care OR healthcare OR (Health ADJ1 Care)) ADJ1 Qualit*) OR ((Institutional OR Protocol OR Policy OR Guideline) ADJ1 (adherence OR Compliance)) OR "Outcome and Process Assessment" OR (Structure ADJ1 Process ADJ1 Outcome ADJ1 Triad*) OR (Donabedian ADJ1 (Model* OR Triad*)) OR (Outcome* ADJ1 (Assessment* OR Research* OR Stud* OR Measure*)) OR (Failure* ADJ1 to ADJ1 Rescue*) OR (Patient ADJ1 Outcome* ADJ1 Assessment*) OR (Patient ADJ1 (Centered OR centred) ADJ1 Outcome* ADJ1 Research) OR (((Patient ADJ1 Relevant) OR Rehabilitation OR Treatment OR (Patient ADJ1 Reported) OR (Critical ADJ1 Care)) ADJ1 Outcome*) OR ((Treatment OR Clinical) ADJ1 (Effectiveness* OR Efficac*)) OR (Treatment ADJ1 Failure*) OR (Process ADJ1 (Assessment* OR Measure*)) OR (((Professional ADJ1 Review*) OR (Peer ADJ1 Review*) OR (Professional ADJ1 Standards ADJ1 Review*)) ADJ1 Organization*) OR PSRO OR "Utilization and Quality Control Peer Review Organizations" OR (Program* ADJ1 (Evaluation* OR Sustainabilit* OR Effectiveness OR Appropriateness)) OR (Best ADJ1 Practice ADJ1 Analysis) OR Benchmark* OR (((Health ADJ1 Care ADJ1 Quality) OR (Healthcare ADJ1 Quality)) ADJ1 (Assurance* OR Assessment*)) OR (Alert ADJ1 Fatigue ADJ1 Health ADJ1 Personnel) OR (Laboratory ADJ1 Proficiency ADJ1 Test*) OR (Close ADJ1 Call*) OR (PIM ADJ1 List*) OR (Potentially ADJ1 Inappropriate ADJ1 Medication*) OR (Beers ADJ1 Criteria*) OR (Beers ADJ1 Potentially ADJ1 Inappropriate ADJ1 Medication*) OR STOPP OR (Screening ADJ1 Tool ADJ1 of ADJ1 Older ADJ1 Person* ADJ1 Potentially ADJ1 Inappropriate ADJ1 Prescription*)).ti,ab,kw.

12 Workload/ OR ((Task ADJ1 Performance*) OR (Critical ADJ1 Incident* ADJ1 Techni*) OR workload* OR (work ADJ1 load*)).ti,ab,kw.

13 Job Satisfaction/ OR ((Job OR work) ADJ1 Satisfaction).ti,ab,kw.

14 (effective* OR ineffective*).ti.

15 (efficien*OR inefficien* OR (clinical ADJ1 effective*) OR productiv*).ti,ab,kw.

16 2 OR 3 OR 4 OR 5 OR 6 OR 7 OR 8 OR 9 OR 10 OR 11 OR 12 OR 13 OR 14 OR 15

17 exp controlled clinical trial/ OR ((clini* ADJ1 trial*) OR ((waitlist* OR (wait* and list*)) and (control* OR group)) OR "treatment as usual" OR tau OR (control* ADJ3 (trial* OR study OR studies OR group*)) OR randomized OR randomized OR groups).ti,ab,kw,pt.

18 cohort analysis/ OR (cohort* OR ((Concurrent OR Incidence OR Followup* OR Prospective OR Longitudinal OR Retrospective OR (Follow ADJ1 up*)) ADJ1 (Study OR studies)) OR (Longitudinal ADJ1 Surve*) OR (before ADJ1 after)).ti,ab,kw,pt.

19 ((multi ADJ1 (center* OR centre*) ADJ1 stud*) OR (multicenter* ADJ1 stud*)).ti,ab,kw,pt.

20 17 OR 18 OR 19

21 1 AND 16 AND 20

22 limit 21 to conference abstract status

23 21 NOT 22

Results: 1,090

**PubMed**

#1 “Physician Assistants"[mesh] OR physician assistant*[tiab] OR physician associate[tiab] OR physician associates[tiab] OR physician extender*[tiab] OR physicians assistant*[tiab] OR physicians extender*[tiab] OR physician substitute*[tiab] OR advanced providers[tiab] OR emergency practitioner*[tiab] OR mid level clinicians[tiab] OR mid level health care professionals[tiab] OR mid level health care provider*[tiab] OR mid level health care workers[tiab] OR mid level health professionals[tiab] OR mid level health providers[tiab] OR mid level health workers[tiab] OR mid level healthcare workers[tiab] OR mid level medical workers[tiab] OR mid level personnel[tiab] OR mid level practitioners[tiab] OR mid level professionals[tiab] OR mid level provider[tiab] OR mid level staff[tiab] OR mid level workers[tiab] OR midlevel clinician*[tiab] OR midlevel health care professional*[tiab] OR midlevel health care provider*[tiab] OR midlevel health care[tiab] OR midlevel health provider*[tiab] OR midlevel health worker*[tiab] OR midlevel personnel[tiab] OR midlevel practitioner*[tiab] OR midlevel professional*[tiab] OR midlevel provider*[tiab] OR non physician clinic staff[tiab] OR non physician clinicians[tiab] OR non physician first assistants[tiab] OR non physician health care personnel[tiab] OR non physician health care professionals[tiab] OR non physician health care providers[tiab] OR non physician health care workers[tiab] OR non physician health professionals[tiab] OR non physician health providers[tiab] OR non physician health workers[tiab] OR non physician healthcare professionals[tiab] OR non physician healthcare providers[tiab] OR non physician healthcare workers[tiab] OR non physician medical personnel[tiab] OR non physician personnel[tiab] OR non physician practice staff[tiab] OR non physician primary care providers[tiab] OR non physician professionals[tiab] OR non physician provider[tiab] OR non physician providers[tiab] OR nonphysician clinic*[tiab] OR nonphysician medical personnel[tiab] OR nonphysician personnel[tiab] OR nonphysician practitioner*[tiab] OR nonphysician primary care clinicians[tiab] OR nonphysician primary care providers[tiab] OR nonphysician specialists[tiab] OR nonphysician staff[tiab] OR advanced practice provider*[tiab] OR advance practice provider*[tiab] OR medical extender*[tiab]

#2 "costs and cost analysis"[mesh] OR "cost of illness"[mesh] OR "Health Care Costs"[Mesh] OR "Insurance"[Mesh] OR "Referral and Consultation"[Mesh] OR Budget control*[tiab] OR Budget saving*[tiab] OR Care budget*[tiab] OR care expen*[tiab] OR Care expen*[tiab] OR Care fund*[tiab] OR Care spend*[tiab] OR champus[tiab] OR Claim analysis[tiab] OR Claim review*[tiab] OR Claims Analysis[tiab] OR Claims Review*[tiab] OR Coinsurance*[tiab] OR Competitive Health Plan*[tiab] OR Competitive Medical Plan*[tiab] OR control cost*[tiab] OR Cost allocat*[tiab] OR Cost analy*[tiab] OR Cost apportionment*[tiab] OR Cost benefit*[tiab] OR Cost compar*[tiab] OR Cost contain*[tiab] OR Cost control*[tiab] OR Cost decreas*[tiab] OR Cost effective*[tiab] OR Cost Efficien*[tiab] OR Cost evaluat*[tiab] OR Cost increase*[tiab] OR Cost manag*[tiab] OR Cost minimi*[tiab] OR Cost reduc*[tiab] OR Cost reduction[tiab] OR Cost saving*[tiab] OR Cost sharing[tiab] OR Cost shifting*[tiab] OR Costeffect*[tiab] OR Cost minimisation[tiab] OR Cost minimization[tiab] OR Deductible*[tiab] OR direct cost*[tiab] OR Economic evaluat*[tiab] OR Health Benefit Plan*[tiab] OR Health budget*[tiab] OR health care cost*[tiab] OR Health care saving*[tiab] OR health care spending[tiab] OR health cost*[tiab] OR health expen*[tiab] OR health expenditure*[tiab] OR Health fund*[tiab] OR Health spend*[tiab] OR health spending*[tiab] OR Healthcare budget*[tiab] OR Healthcare cost*[tiab] OR healthcare expen*[tiab] OR Healthcare fund*[tiab] OR Healthcare savings[tiab] OR Healthcare spend*[tiab] OR healthcare spending*[tiab] OR High cost*[tiab] OR High spend*[tiab] OR Increasing cost*[tiab] OR insuran*[tiab] OR Low cost*[tiab] OR managed car*[tiab] OR Medical budget*[tiab] OR Medical Care Cost*[tiab] OR medical cost*[tiab] OR Medical expen*[tiab] OR Medical fund*[tiab] OR medical saving*[tiab] OR Medical saving*[tiab] OR Medical spend*[tiab] OR medicare[tiab] OR Preferred provider*[tiab] OR Reducing cost*[tiab] OR Reimburs*[tiab] OR Rising cost*[tiab] OR Saving cost*[tiab] OR societal cost*[tiab] OR Third-Party Pay*[tiab] OR Treatment Cost*[tiab] OR Usage reduction*[tiab] OR Value Based Purchas*[tiab] OR Worker Compensation*[tiab] OR Worker s compensation*[tiab] OR Workers compensation*[tiab] OR Return on investment*[tiab] OR ROI[tiab]

#3 "Waiting Lists"[Mesh] OR waiting list*[tiab] OR waitlist*[tiab] OR waitlist*[tiab] OR delist*[tiab] OR wait period*[tiab] OR waiting period*[tiab] OR waiting time*[tiab] OR wait time*[tiab]

#4 "Hospitalization"[Mesh] OR Hospitaliz*[tiab] OR Hospitalis*[tiab] OR Admission*[tiab] OR Discharg*[tiab] OR Handoff*[tiab] OR Hand Over*[tiab] OR Sign Out*[tiab] OR Signout*[tiab] OR Hand Off*[tiab] OR Handover*[tiab] OR Readmis*[tiab] OR Patient Transfer*[tiab] OR Patient Transition*[tiab] OR Care Transition*[tiab] OR Transition of Care*[tiab] OR Health Care Transition*[tiab] OR Healthcare transition*[tiab] OR Patient Turfing*[tiab] OR Patient Dumping*[tiab] OR length of stay*[tiab] OR Stay Length[tiab] OR Hospital Stay*[tiab] OR duration of stay*[tiab] OR lengths of stay*[tiab]

#5 "Mortality"[Mesh] OR "mortality" [Subheading] OR Mortalit*[tiab] OR Case Fatality Rate*[tiab] OR Death Rate*[tiab] OR Fatal Outcome*[tiab]

#6 "Morbidity"[Mesh] OR morbidit*[tiab] OR comorbidit*[tiab]

#7 "Quality of Life"[Mesh] OR "Health Status"[Mesh] OR "Activities of Daily Living"[Mesh] OR "Quality-Adjusted Life Years"[Mesh] OR Quality Adjusted Life Years[tiab] OR QALY[tiab] OR Healthy Years Equivalent*[tiab] OR Adjusted Life Year*[tiab] OR life qualit*[tiab] OR quality of life[tiab] OR health status[tiab] OR level of health[tiab] OR health level*[tiab] OR qol[tiab] OR hrql[tiab] OR hrqol[tiab] OR activities of daily living[tiab] OR daily living activit*[tiab] OR adl[tiab] OR chronic limitation of activit*[tiab]

#8 "Patient Satisfaction"[Mesh] OR Patient Satisfaction*[tiab] OR Patient preference*[tiab] OR patient experienc*[tiab] OR satisfaction of patient*[tiab]

#9 "Patient Compliance"[Mesh:NoExp] OR "Medication Adherence"[Mesh] OR Patient Adher*[tiab] OR Patient Cooperat*[tiab] OR Patient Non-Compli*[tiab] OR Patient Nonadher*[tiab] Patient Noncompli*[tiab] OR Patient Non Adher*[tiab] OR Treatment Complianc*[tiab] OR Therapeutic Complianc*[tiab] OR Medication Nonadher*[tiab] OR Medication Noncomplianc*[tiab] OR Medication Non Adher*[tiab] OR Medication Persistence*[tiab] OR Medication Complianc*[tiab] OR Medication Non Complianc*[tiab]

#10 "Patient Safety"[Mesh] OR "Medical Errors"[Mesh] OR Medical Mistake*[tiab] OR Medical Error*[tiab] OR Wrong-Procedure Error*[tiab] OR Wrong-Site Surger*[tiab] OR Surgical Error*[tiab] OR Critical Medical Incident*[tiab] OR Never Event*[tiab] OR Diagnostic Error*[tiab] OR Misdiagnos*[tiab] OR False Negative Reaction*[tiab] OR False Positive Reaction*[tiab] OR Observer Variation*[tiab] OR Observer Bias[tiab] OR Interobserver Variation*[tiab] OR Inter-Observer Variation*[tiab] OR Interobserver Variabilit*[tiab] OR Inter-Observer Variabilit*[tiab] OR Intraobserver Variation*[tiab] OR Intra-Observer Variation*[tiab] OR Intraobserver Variabilit*[tiab] OR Intra Observer Variabilit*[tiab] OR Near Miss*[tiab] OR Close Call*[tiab] OR Radiotherapy Setup Error*[tiab] OR Medication Error*[tiab] OR Drug Use Error*[tiab] OR patient safet*[tiab]

#11 "Quality of Health Care"[mesh] OR Health Care Qualit*[tiab] OR Quality of Healthcare[tiab] OR Healthcare Quality[tiab] OR Quality of Care[tiab] OR Care Qualit*[tiab] OR Guideline adherence[tiab] OR Policy Compliance[tiab] OR Protocol Compliance[tiab] OR Institutional Adherence[tiab] OR “Outcome and Process Assessment”[tiab] OR Structure Process Outcome Triad*[tiab] OR Donabedian Model[tiab] OR Donabedian Triad[tiab] OR Outcomes Assessment*[tiab] OR Outcome Assessment*[tiab] OR Outcomes Research[tiab] OR Outcome Stud*[tiab] OR Outcome Measure*[tiab] OR Failure to Rescue*[tiab] OR Failures to Rescue*[tiab] OR Patient Outcome Assessment*[tiab] OR Patient Centered Outcomes Research[tiab] OR Critical Care Outcome*[tiab] OR Patient Reported Outcome*[tiab] OR Treatment outcome*[tiab] OR Patient Relevant Outcome*[tiab] OR Clinical Efficac*[tiab] OR Treatment Effectiveness[tiab] OR Treatment Efficac*[tiab] OR Rehabilitation Outcome*[tiab] OR Treatment Failure*[tiab] OR Process Assessment*[tiab] OR Process Measure*[tiab] OR Professional Review Organization*[tiab] OR Professional Standards Review Organization*[tiab] OR PSRO[tiab] OR Peer Review Organization*[tiab] OR “Utilization and Quality Control Peer Review Organizations”[tiab] OR Program Evaluation*[tiab] OR Program Sustainabilit*[tiab] OR Program Effectiveness[tiab] OR Program Appropriateness[tiab] OR Best Practice Analysis[tiab] OR Benchmark*[tiab] OR Healthcare Quality Assurance*[tiab] OR Health Care Quality Assurance*[tiab] OR Healthcare Quality Assessment*[tiab] OR Health Care Quality Assessment*[tiab] OR Alert Fatigue Health Personnel[tiab] OR Laboratory Proficiency Test*[tiab] OR Near Miss*[tiab] OR Close Call*[tiab] OR PIM List*[tiab] OR Potentially Inappropriate Medication*[tiab] OR Beers Criteria*[tiab] OR Beers Potentially Inappropriate Medications[tiab] OR STOPP[tiab] OR Screening Tool of Older Person's Potentially Inappropriate Prescription*[tiab]

#12 "Workload"[Mesh] OR "Task Performance and Analysis"[Mesh] OR Task Performance*[tiab] OR Critical Incident Techni*[tiab] OR workload*[tiab] OR work load*[tiab]

#13 "Job Satisfaction"[Mesh] OR Job Satisfaction[tiab] OR work Satisfaction[tiab]

#14 Efficiency[mesh] OR efficien*[tiab] OR inefficien*[tiab] OR clinical effective*[tiab] OR productiv*[tiab] OR effective*[ti] OR ineffective*[ti]

#15 #2 OR #3 OR #4 OR #5 OR #6 OR #7 OR #8 OR #9 OR #10 OR #11 OR #12 OR #13 OR #14

#16 randomized controlled trial[pt] OR controlled clinical trial[pt] OR randomized[tiab] OR randomised[tiab] OR drug therapy[sh] OR randomly[tiab] OR trial[tiab] OR groups[tiab]

#17 "Cohort Studies"[Mesh] OR cohort*[tiab] OR Concurrent Stud*[tiab] OR Incidence Stud*[tiab] OR Followup Stud*[tiab] OR Follow up Stud*[tiab] OR Longitudinal Stud*[tiab] OR Longitudinal Surve*[tiab] OR Prospective Stud*[tiab] OR Retrospective Stud*[tiab] OR before after[tiab]

#18 multi center stud*[tiab] OR multi centre stud*[tiab] OR multicenter stud*[tiab] OR multi centre stud*[tiab]

#19 #16 OR #17 OR #18

#20 #1 AND #15 AND #19

Results: 1,657

**WOS**

#1 TS=((“physician” NEAR/1 (assistant* OR “associate” OR “associates” OR extender* OR substitute*)) OR (medical NEAR/1 extender*) OR (advance* NEAR/1 provider*) OR (“emergency” NEAR/0 practitioner*) OR ((“Midlevel” OR (“mid” NEAR/1 “level”) OR ((“non” OR “none”) NEAR/1 physician*) OR nonphysician*) NEAR/2 (clinician* OR ((“health” OR “healthcare”) NEAR/2 (professional* OR provider* OR worker*)) OR “personnel” OR practitioner* OR professional* OR provider* OR “staff” OR worker* OR specialist* OR (“first” NEAR/1 assistant*))) OR (“midlevel” NEAR/1 “health” NEAR/1 “care”) OR (advance* NEAR/1 “practice” NEAR/1 provider*))

#2 TS=(((Spend* OR Fund* OR Expen* OR Budget*) NEAR/1 (control* OR saving* OR “care” OR health* OR “high” OR “medical”)) OR “champus” OR (Claim* NEAR/1 (“analysis” OR review*)) OR Coinsurance* OR (“Competitive” NEAR/1 (Health* OR “medical”) NEAR/1 Plan*) OR Costeffect* OR Deductible* OR (“direct” NEAR/1 cost*) OR (“Economic” NEAR/1 evaluat*) OR (“Health” NEAR/1 Benefit* NEAR/1 Plan*) OR insuran* OR (“managed” NEAR/1 car*) OR “medicare” OR (“Preferred” NEAR/1 provider*) OR Reimburs* OR (“Third” NEAR/1 “Party” NEAR/1 Pay*) OR (“Usage” NEAR/1 reduction*) OR (“Value” NEAR/1 “Based” NEAR/1 Purchas*) OR (Worker* NEAR/1 Compensation*) OR (“Return” NEAR/1 “on” NEAR/1 investment*) OR “ROI” OR ((“Cost” OR “costs”) NEAR/1 (allocat* OR analy* OR apportionment* OR benefit* OR compar* OR contain* OR control* OR decreas* OR effective* OR Efficien* OR evaluat* OR increase* OR manag* OR minimi* OR reduc* OR saving* OR “sharing” OR shifting* OR “minimization” OR “minimization” OR (“health” NEAR/1 “care”) OR health* OR high* OR low* OR increas* OR “medical” OR “rising” OR “societal” OR “Treatment”)))

#3 TS=(waitlist* OR delist* OR (wait* NEAR/1 (“period” OR list* OR “time”)))

#4 TS=(Hospitaliz* OR Hospitalis* OR Admission* OR Discharg* OR Handoff* OR (Hand* NEAR/1 (Over* OR “off”)) OR (“Sign” NEAR/1 Out*) OR Signout* OR Handover* OR Readmis* OR ((“healthcare” OR “care” OR patient*) NEAR/1 (transfer* OR transition*)) OR (Patient* NEAR/1 (Turfing* OR Dumping*)) OR ((“Duration” OR “Hospital” OR Length*) NEAR/1 stay*))

#5 TS=(Mortalit* OR (“Case” NEAR/1 “Fatality” NEAR/1 Rate*) OR (“Death” NEAR/1 Rate*) OR (“Fatal” NEAR/1 Outcome*))

#6 TS=(morbidit* OR comorbidit*)

#7 TS=((“Quality” NEAR/1 “Adjusted” NEAR/1 Years) OR “QALY” OR (“Healthy” NEAR/1 Year* NEAR/1 Equivalent*) OR (“Adjusted” NEAR/1 “Life” NEAR/1 Year*) OR (“life” NEAR/1 qualit*) OR (“quality” NEAR/1 “of” NEAR/1 “life”) OR (“health” NEAR/1 “status”) OR (“level” NEAR/1 “of” NEAR/1 “health”) OR (“health” NEAR/1 level*) OR “qol” OR “hrql” OR “hrqol” OR (activit* NEAR/1 “of” NEAR/1 “daily” NEAR/1 “living”) OR (“daily” NEAR/1 activit*) OR “adl” OR (“chronic” NEAR/1 “limitation” NEAR/1 “of” NEAR/1 activit*))

#8 TS=(Patient* NEAR/1 (Satisfaction* OR preference* OR experienc*))

#9 TS=((“medication” OR “Therapeutic” OR “Treatment” OR Patient*) NEAR/1 (Adher* OR Cooperat* OR ((“Non” OR “none”) NEAR/1 (Compli* OR adher*)) OR Nonadher* OR Noncompli*))

#10 TS=((“Wrong” NEAR/1 Procedure*) OR (“Wrong” NEAR/1 “Site” NEAR/1 Surger*) OR ((“Surgical” OR “Medical” OR “Diagnostic”) NEAR/1 (Mistake* OR Error*)) OR (“Critical” NEAR/1 “Medical” NEAR/1 Incident*) OR (“Never” NEAR/1 Event*) OR Misdiagnos* OR (“False” NEAR/1 (“Negative” OR “positive”) NEAR/1 Reaction*) OR ((“Intraobserver” OR “Interobserver” OR Observer*) NEAR/1 (Variation* OR “bias” OR Variabilit*)) OR (“Near” NEAR/1 Miss*) OR (“Close” NEAR/1 Call*) OR (“Radiotherapy” NEAR/1 “Setup” NEAR/1 Error*) OR ((“Medication” OR (“Drug” NEAR/1 “Use”)) NEAR/1 Error*) OR (patient* NEAR/1 safet*))

#11 TS=(((“care” OR “healthcare” OR (“Health” NEAR/1 “Care”)) NEAR/1 Qualit*) OR ((“Institutional” OR “Protocol” OR “Policy” OR “Guideline”) NEAR/1 (“adherence” OR “Compliance”)) OR “Outcome and Process Assessment” OR (“Structure” NEAR/1 “Process” NEAR/1 “Outcome” NEAR/1 Triad*) OR (“Donabedian” NEAR/1 (Model* OR Triad*)) OR (Outcome* NEAR/1 (Assessment* OR Research* OR Stud* OR Measure*)) OR (Failure* NEAR/1 “to” NEAR/1 Rescue*) OR (“Patient” NEAR/1 Outcome* NEAR/1 Assessment*) OR (“Patient” NEAR/1 (“Centered” OR “centred”) NEAR/1 Outcome* NEAR/1 “Research”) OR (((“Patient” NEAR/1 “Relevant”) OR “Rehabilitation” OR “Treatment” OR (“Patient” NEAR/1 “Reported”) OR (“Critical” NEAR/1 “Care”)) NEAR/1 Outcome*) OR ((“Treatment” OR “Clinical”) NEAR/1 (Effectiveness* OR Efficac*)) OR (“Treatment” NEAR/1 Failure*) OR (“Process” NEAR/1 (Assessment* OR Measure*)) OR (((“Professional” NEAR/1 Review*) OR (“Peer” NEAR/1 Review*) OR (“Professional” NEAR/1 “Standards” NEAR/1 Review*)) NEAR/1 Organization*) OR “PSRO” OR “Utilization and Quality Control Peer Review Organizations” OR (Program* NEAR/1 (Evaluation* OR Sustainabilit* OR “Effectiveness” OR “Appropriateness”)) OR (“Best” NEAR/1 “Practice” NEAR/1 “Analysis”) OR Benchmark* OR (((“Health” NEAR/1 “Care” NEAR/1 “Quality”) OR (“Healthcare” NEAR/1 “Quality”)) NEAR/1 (Assurance* OR Assessment*)) OR (“Alert” NEAR/1 “Fatigue” NEAR/1 “Health” NEAR/1 “Personnel”) OR (“Laboratory” NEAR/1 “Proficiency” NEAR/1 Test*) OR (“Close” NEAR/1 Call*) OR (“PIM” NEAR/1 List*) OR (“Potentially” NEAR/1 “Inappropriate” NEAR/1 Medication*) OR (“Beers” NEAR/1 Criteria*) OR (“Beers” NEAR/1 “Potentially” NEAR/1 “Inappropriate” NEAR/1 Medication*) OR “STOPP” OR (“Screening” NEAR/1 “Tool” NEAR/1 “of” NEAR/1 “Older” NEAR/1 Person* NEAR/1 “Potentially” NEAR/1 “Inappropriate” NEAR/1 Prescription*))

#12 TS=((“Task” NEAR/1 Performance*) OR (“Critical” NEAR/1 Incident* NEAR/1 Techni*) OR workload* OR (“work” NEAR/1 load*))

#13 TS=((“Job” OR “work”) NEAR/1 “Satisfaction”)

#14 #2 OR #3 OR #4 OR #5 OR #6 OR #7 OR #8 OR #9 OR #10 OR #11 OR #12 OR #13

#15 TS=((clini* NEAR/1 trial*) OR (singl* NEAR/1 blind*) OR (singl* NEAR/1 mask*) OR (doubl* NEAR/1 blind*) OR (doubl* NEAR/1 mask*) OR (tripl* NEAR/1 blind*) OR (tripl* NEAR/1 mask*) OR (random* NEAR/1 allocat*) OR placebo* OR ((waitlist* OR (wait* and list*)) and (control* OR “group”)) OR "treatment as usual" OR “tau” OR (control* N3 (trial* OR “study” OR “studies” OR group*)) OR “randomized” OR “randomized”)

#16 TS=(cohort* OR ((“Concurrent” OR “Incidence” OR Followup* OR “Prospective” OR “Longitudinal” OR “Retrospective” OR (“Follow” NEAR/1 “up”)) NEAR/1 (“Study” OR “studies”)) OR (“Longitudinal” NEAR/1 Surve*) OR (“before” NEAR/1 “after”))

#17 TS=((“multi” NEAR/1 (center* OR centre*) NEAR/1 stud*) OR (multicenter* NEAR/1 stud*))

#18 #15 OR #16 OR #17

#19 #1 AND #14 AND #18

#20 TS=(efficien*OR inefficien* OR (“clinical” NEAR/1 effective*) OR productiv* OR effective* OR ineffective*)

#21 #19 OR #21

Results: 524
